# Prediction of recurrent venous thrombosis in all patients with a first venous thrombotic event: The Leiden Thrombosis Recurrence Risk Prediction model (L-TRRiP)

**DOI:** 10.1371/journal.pmed.1002883

**Published:** 2019-10-11

**Authors:** Jasmijn F. Timp, Sigrid K. Braekkan, Willem M. Lijfering, Astrid van Hylckama Vlieg, John-Bjarne Hansen, Frits R. Rosendaal, Saskia le Cessie, Suzanne C. Cannegieter

**Affiliations:** 1 Department of Clinical Epidemiology, Leiden University Medical Center, Leiden, the Netherlands; 2 K. G. Jebsen Thrombosis Research and Expertise Center (TREC), Department of Clinical Medicine, UiT–The Arctic University of Norway, Tromsø, Norway; 3 Division of Internal Medicine, University Hospital of North Norway, Tromsø, Norway; 4 Department of Medical Statistics, Leiden University Medical Center, Leiden, the Netherlands; 5 Department of Internal Medicine, Section Thrombosis and Hemostasis, Leiden University Medical Center, Leiden, the Netherlands; University of Oxford, UNITED KINGDOM

## Abstract

**Background:**

Recurrent venous thromboembolism (VTE) is common. Current guidelines suggest that patients with unprovoked VTE should continue anticoagulants unless they have a high bleeding risk, whereas all others can stop. Prediction models may refine this dichotomous distinction, but existing models apply only to patients with unprovoked first thrombosis. We aimed to develop a prediction model for all patients with first VTE, either provoked or unprovoked.

**Methods and findings:**

Data were used from two population-based cohorts of patients with first VTE from the Netherlands (Multiple Environment and Genetic Assessment of Risk Factors for Venous Thrombosis [MEGA] follow-up study, performed from 1994 to 2009; model derivation; *n* = 3,750) and from Norway (Tromsø study, performed from 1999 to 2016; model validation; *n* = 663). Four versions of a VTE prediction model were developed: model A (clinical, laboratory, and genetic variables), model B (clinical variables and fewer laboratory markers), model C (clinical and genetic factors), and model D (clinical variables only). The outcome measure was recurrent VTE. To determine the discriminatory power, Harrell’s C-statistic was calculated. A prognostic score was assessed for each patient. Kaplan-Meier plots for the observed recurrence risks were created in quintiles of the prognostic scores. For each patient, the 2-year predicted recurrence risk was calculated. Models C and D were validated in the Tromsø study.

During 19,201 person-years of follow-up (median duration 5.7 years) in the MEGA study, 507 recurrences occurred. Model A had the highest predictive capability, with a C-statistic of 0.73 (95% CI 0.71–0.76). The discriminative performance was somewhat lower in the other models, with C-statistics of 0.72 for model B, 0.70 for model C, and 0.69 for model D. Internal validation showed a minimal degree of optimism bias. Models C and D were externally validated, with C-statistics of 0.64 (95% CI 0.62–0.66) and 0.65 (95% CI 0.63–0.66), respectively. According to model C, in 2,592 patients with provoked first events, 367 (15%) patients had a predicted 2-year risk of >10%, whereas in 1,082 patients whose first event was unprovoked, 484 (45%) had a predicted 2-year risk of <10%. A limitation of both cohorts is that laboratory measurements were missing in a substantial proportion of patients, which therefore were imputed.

**Conclusions:**

The prediction model we propose applies to patients with provoked or unprovoked first VTE—except for patients with (a history of) cancer—allows refined risk stratification, and is easily usable. For optimal individualized treatment, a management study in which bleeding risks are also taken into account is necessary.

## Abstract 2

Suzanne C. Cannegieter and colleagues present a model for predicting repeat venous thromboembolism.

## Author summary

**Why was this study done?:**

- Patients who suffered from a deep vein thrombosis or pulmonary embolism for the first time have an average risk of 3%–5% per year of having a second event.
- Such a recurrence can be prevented with anticoagulant treatment, but this should be continued indefinitely, which will lead to a substantial risk of bleeding.
- The risks of both recurrent thrombosis and bleeding differ substantially between individuals.
- The choice of continuation on the one hand (with a decreased thrombosis risk but an increased bleeding risk) and discontinuation on the other hand (with an increased thrombosis risk but a decreased bleeding risk) is currently difficult to make for both clinicians and patients. Individual differences in risks are hardly taken into account.

**What did the researchers do and find?:**

- Data were used from two population-based cohorts of patients with first venous thrombosis from the Netherlands (Multiple Environment and Genetic Assessment of Risk Factors for Venous Thrombosis [MEGA] study; *n* = 3,750) and from Norway (Tromsø study; *n* = 663).
- Four versions of a venous thromboembolism (VTE) recurrence risk prediction model were developed in the MEGA data, differing in number and type of included predictors, of which two were validated in the Tromsø study.
- During 19,201 person-years of follow-up, 507 recurrences occurred. The predictive capability varied between the models.
- A substantial proportion of patients with a first venous thrombosis are currently misclassified with respect to duration of anticoagulant treatment.

**What do these findings mean?:**

- The prediction model we propose applies to all patients, allows refined risk stratification, and is easily usable.
- A limitation of both cohorts is that laboratory measurements were missing in a substantial proportion of patients, which were imputed.
- For optimal individualized treatment, a management study is necessary in which bleeding risks are also taken into account.

## Introduction

Recurrent venous thromboembolism (VTE) after a first deep vein thrombosis (DVT) or pulmonary embolism (PE) is common, with a 5-year cumulative incidence of approximately 25% [1,2]. The primary treatment of VTE consists of anticoagulants for a limited period of time, generally 3–6 months [3]. This period serves as treatment of the acute episode to prevent extension or (further) embolization of the thrombus [4]. Secondary prevention by means of continuation of the anticoagulant treatment will lead to reduction in the number of recurrent events [5]. However, the decision to continue treatment is challenging in the clinic, as it has strong, lifelong implications. The risks of recurrence when treatment is stopped and of bleeding when treatment is continued are high and persist over a patient’s lifetime [6,7]. For this, an accurate assessment of both risks is important.

Current guidelines generally advise patients with a provoked first VTE to discontinue treatment after 3 months and patients with an unprovoked first event to continue (taking risk of bleeding and patient preferences into account) [3,8]. One problem with this approach is classifying an event as unprovoked in routine clinical practice, although no unequivocal definition of such events is available, and discussion remains on risk factors like oral contraceptive use, outpatient immobilization, prolonged travel, and thrombophilia [9–12]. Furthermore, we previously showed that this dichotomized approach is too crude because the absolute risks of recurrence in patients vary considerably within these groups [9,13]. Hence, patients will be either over- or undertreated.

An alternative for the situation as advised in the current guidelines is to classify patients in a more refined way, according to the presence of other risk factors. Although some predictive factors for recurrent VTE have been identified, such as the type of the first event, male sex, and the presence of an active malignancy [14], none of these factors has enough distinctive power on their own to classify patients into high or low risk of recurrence. A prediction model combining several factors is needed for this purpose. The three best-known prediction models for recurrent VTE are (1) the “men continue and HERDOO2 rule,” (2) the Vienna prediction model, and (3) the D-dimer, Age, Sex, Hormonal therapy (DASH) score [15–17]. Although their predictive performances are acceptable [18–20], they suffer from some limitations [9,10,13]. First of all, they were developed only for patients with a first unprovoked VTE [15–17]. Because of the lack of unequivocal criteria for unprovoked VTE, definitions vary considerably in the three models, which leads to differing unprovoked patient groups. We showed in an external validation study that the predictive performance of these models drops when a different definition of unprovoked thrombosis is applied [13]. A model without this requirement would be easier to apply in daily practice and lead to more accurate predictions. A second limitation of the current models is that they aimed to make simple scores to facilitate their use in practice. Although this is obviously a laudable objective, simple scores also lead to reduced discriminatory performance. Besides, the current common use of “apps” in clinical practice has outdated the concern of complicated scores: a user-friendly app or an algorithm in electronic patient records can directly provide a patient’s risk score. This allows more complicated models with the use of more predictive variables, which can much improve discriminatory performance [21]. A third limitation of the current models is that all recurrent events were considered as outcome events in their derivation, whereas it would be preferable to leave out the recurrences that occurred in high-risk situations such as surgery. For these events, the specific situation (e.g., surgery, pregnancy, plaster cast) is the major causative factor, the occurrence of which is hard to predict by factors assessed at the time of the initial event [22]. Moreover, patients should receive thromboprophylaxis in these situations, which will make such recurrences even more unpredictable.

We aimed to develop a prediction model for all patients with first VTE, either provoked or unprovoked, building on the currently available models but with inclusion of a large set of both clinical and laboratory candidate predictor parameters.

## Methods

### Patient population

Between March 1999 and August 2004, 4,956 patients aged 18–70 years with an objectively diagnosed first DVT of the leg or PE were included in a population-based case-control study (Multiple Environment and Genetic Assessment of Risk Factors for Venous Thrombosis [MEGA] study). All patients filled in an extensive questionnaire on putative risk factors for VTE. Blood samples were collected at least 3 months after discontinuation of anticoagulant treatment. Details of the MEGA study have been described previously; for the MEGA protocol, please see S1 Protocol [23,24].

Of the MEGA case-control study, only the cases were further followed for recurrence (MEGA follow-up study). For this, 225 of the 4,956 patients did not consent, leaving 4,731 patients. Between 2007 and 2009, the vital status of all patients was acquired from the central Dutch population register [25], and for the patients who died, a cause of death (ICD-10-CM) was obtained from the national register of death certificates at the Central Bureau of Statistics. Short-answer forms concerning recurrent VTE were sent by mail to all survivors and consenting individuals between June 2008 and July 2009 and were supplemented by telephone interviews. Furthermore, all patients were asked to complete a second questionnaire on the presence of risk factors for VTE after their first event [26]. This study was approved by the Medical Ethics Committee of the Leiden University Medical Center, and all participants gave written informed consent.

### Recurrent venous thrombosis

During the same period, when patients were asked to self-report on any recurrent thrombotic events, additional information about recurrences was retrieved from the anticoagulation clinics where patients were initially included for their first event and, in case they moved house, at the clinic nearest to their new address. Death due to VTE was also included. For recurrent events reported by the patient or the clinic, discharge letters from the treating physician were obtained, including information from objective diagnostic procedures. An adjudication rule regarding certainty of the diagnosis was applied using the information collected per patient (see S1 Text [26]). According to this rule, possible recurrences were classified into certain recurrences and uncertain recurrences to distinguish truly new thromboses from extensions of a first event. For this study, we considered certain recurrences in the absence of a high-risk situation as outcome event (defined as surgery, trauma, plaster cast, pregnancy, immobilization, malignancy, or hormone use during the follow-up period). Unprovoked first VTE was defined as VTE without surgery, trauma, plaster cast, pregnancy, or immobilization in the first 3 months before the event; active malignancies (in the 5 years prior to the first event); hormone use (oral contraceptives or hormone replacement therapy); or prolonged travel at the time of the event. Patients who had one or more of these risk factors at time of their first event were classified as having a provoked VTE. Patients with uncertain recurrent events or events that occurred in a high-risk situation were censored from this recurrent event onward.

### Blood sampling and laboratory analyses

Approximately 3 months after discontinuation of oral anticoagulant therapy, patients were invited for collection of a blood sample unless they were still on anticoagulant therapy 1 year after their event, in which case blood was drawn during treatment [23]. Blood sampling was requested until June 2002 for logistic reasons, and thereafter, patients were sent buccal swabs to collect DNA. Blood samples were drawn into vacuum tubes containing 0.1 volume 0.106 mol/L trisodium citrate and centrifuged for 10 minutes at 4°C, after which plasma was aliquoted, frozen, and stored at −80°C. Assays for factor VIII (FVIII), von Willebrand factor, D-dimer, FVII, factor V (FV), factor X (FX), free protein S, protein C activity, fibrinogen, FII, FIX, FXI, TFPI, antithrombin, hemoglobin, white blood cell count, monocyte percentage, red cell distribution width, high-sensitivity C-reactive protein (CRP), activated protein C resistance (APCsr), and thrombin generation (ETP) were performed in automated machines by laboratory technicians, as well as the assays for five single nucleotide polymorphisms (SNPs) that have been associated with VTE risk: rs6025 (F5, FV Leiden), rs1799963 (F2, 20210 G>A), rs8176719 (ABO blood group), rs2066865 (FGG 10,034 C>T), and rs2036914 (F11) [27]. See supporting file S1 Text for details on the laboratory analyses.

### Current analysis: Follow-up and patients included

**Fig 1:**
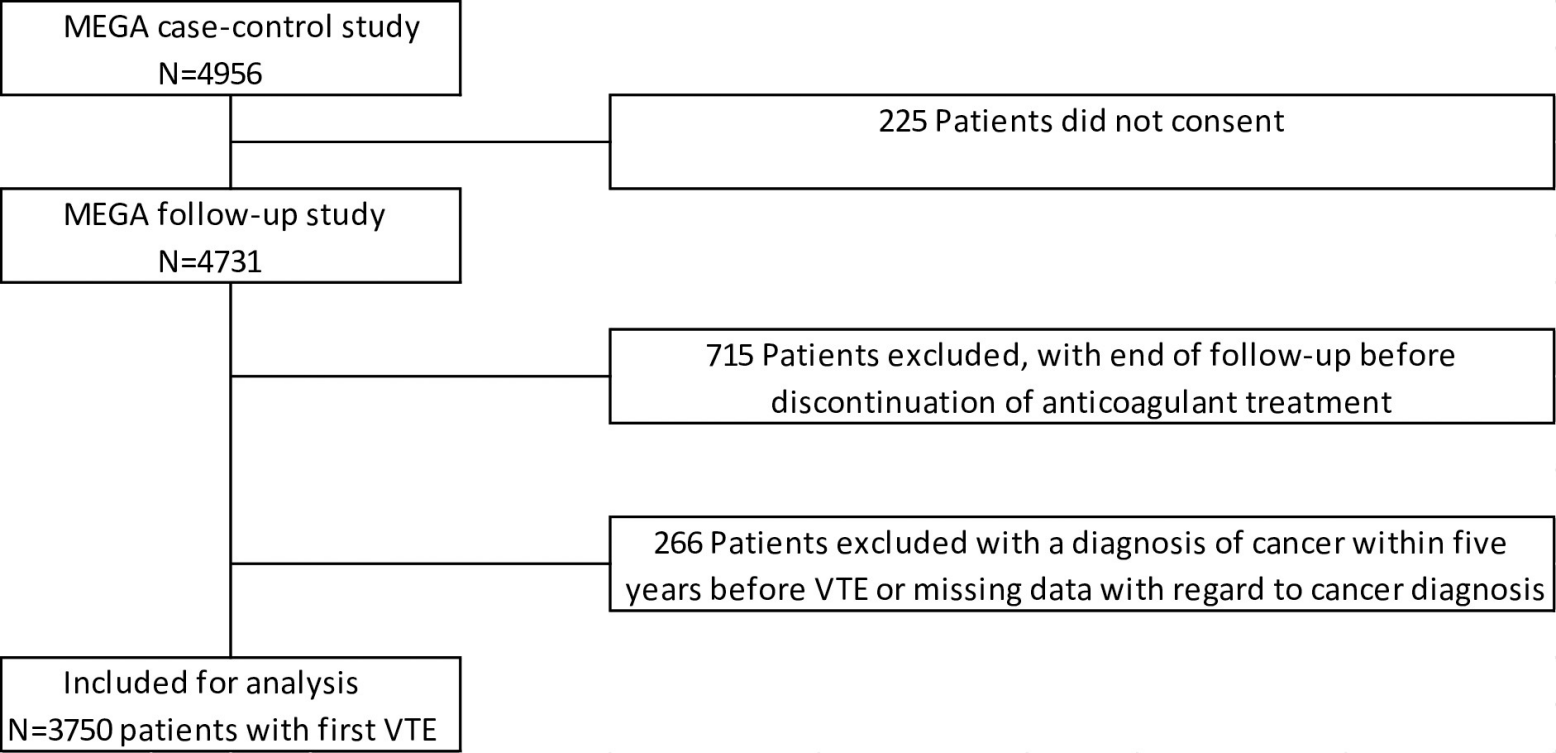
Flowchart of included and excluded patients from analyses. MEGA; Multiple Environment and Genetic Assessment of Risk Factors for Venous Thrombosis; VTE, venous thromboembolism.

Follow-up started at the moment of discontinuation of anticoagulant treatment of the index event (so generally after 3–6 months) because this is the point in time when a decision needs to be made on continuation of the treatment. Hence, patients who were on continuous anticoagulant treatment were excluded. The end of follow-up was defined as the date of a recurrence or, in its absence, the date of returning the short-answer forms. The last form was returned on April 8, 2010. If patients did not complete the form, they were censored at the last date we knew them to be recurrence free (date of death [*n* = 22], date of emigration [*n* = 3], date last seen by the anticoagulation clinic or for research purposes [*n* = 354]). In total, 715 patients were excluded from the MEGA follow-up study because their follow-up ended before or at the moment of discontinuation of anticoagulant treatment, so these patients either continued treatment (*n* = 575), died (*n* = 53), or had a recurrence during anticoagulant treatment (*n* = 87; of which *n* = 37 were classified as certain recurrences). Furthermore, we chose to exclude patients with a history of cancer because specific guidelines exist for this particular patient group [3,27]. Hence, 266 patients were excluded who had a diagnosis of cancer within 5 years before VTE or in whom data on a possible cancer diagnosis in the past were missing. In total, 43 recurrences occurred in the 3-month time period between discontinuation of anticoagulation and blood sampling. These patients were included in the analysis because follow-up started at time of discontinuation of anticoagulation. See Fig 1 for a flowchart with the number of patients included (*n* = 3,750) and excluded (*n* = 981) for analyses. Furthermore, patients with a provoked certain recurrent event were censored (*n* = 93), as well as 145 patients with uncertain recurrent events.

### Missing values

For logistic reasons, blood sampling was performed until June 2002, until which time approximately 50% of patients in the MEGA study were included [26,28]. For 2,107 patients out of 3,750 (56%), blood samples and measurements on laboratory markers were available. Multiple imputation techniques were used for missing values on these measurements. The same was done for vitamin K–dependent coagulation factor levels in participants whose blood was drawn during anticoagulant treatment (*n* = 190). Furthermore, all other predictor variables that were missing were imputed. Detailed information on missing data can be found in S1 Table. In the imputation step, skewed variables were log-transformed. In all, 10 datasets were imputed, and results were pooled according to Rubin’s rules [29].

### Development of the Leiden Thrombosis Recurrence Risk Prediction model

Prediction models were developed on 3,750 patients with a first VTE, either provoked or unprovoked. Identification of candidate predictor variables was based on (1) consistent inclusion in previous prediction models; (2) reported associations with the occurrence of (recurrent) VTE in literature; or (3) expert opinion, leading to a total number of 39 candidate predictors for the most extensive (maximum) model [28]. For an overview of these variables, see S1 Table. Levels of coagulation factors or laboratory markers were entered into the models as continuous variables, whereas most other predictor variables were set as categorical. For the continuous variables, we checked linearity by adding a quadratic term to the models, but we found no evidence for nonlinear associations.

Prediction models were created by means of Cox regression analyses using a backward selection procedure (criterion *p* < 0.15). We developed a maximum model, including all candidate predictor variables (model A); a limited model, including clinical variables and only laboratory markers that are easy to assess in the clinic (model B); a model including clinical and genetic variables only (model C); and a model including clinical variables only (model D). We also considered the current clinical situation by fitting a model with type of first event (provoked or unprovoked) as the only predictor. We checked whether the proportional hazards assumption was met by testing for time by covariate interactions. No evidence of nonproportionality was found, except for pregnancy, but the number of recurrences in women who were pregnant at the first event was very low (4).

To determine the magnitude of discrimination of the model, Harrell’s C-statistic was calculated for the four models [29]. As a sensitivity analysis, we estimated Harrell’s C-statistics also in the complete cases only—i.e., in the nonimputed data. However, for risk prediction, the actual or absolute predicted risk is of more clinical interest. Therefore, the prognostic score for each patient was calculated by beta1*x1 + beta2*x2 + beta3*x3 + …, where the x1, x2, x3, etc., represent the variables in the prediction model, and beta1, beta2, beta3, etc., represent the corresponding estimated regression coefficients. To be able to visualize how well the models are able to distinguish between risks of recurrence, (inverse) Kaplan-Meier plots for the observed recurrence risks were created in quintiles of the prognostic score. For each patient, the 2-year predicted risk of recurrence was calculated with the baseline 2-year recurrence-free probability S_0_ and the prognostic score, using the following equation: risk of recurrence = 1 − S_0_** exp(prognostic score). We created calibration plots by plotting the observed 2-year risks in the quintiles of the prognostic score against the mean 2-year risks as estimated by the four models.

For clinical decision-making, the absolute predicted risk is relevant. According to the International Society on Thrombosis and Haemostasis (ISTH), patients who have a yearly absolute risk of (either provoked or unprovoked) recurrent VTE of >5% are candidates to receive secondary prevention by prolonged treatment with oral anticoagulant therapy [30]. Therefore, we depicted the distribution of individual predicted 2-year recurrence risks and determined the proportions of patients above and below this threshold of 10%.

### Internal validation of prediction models

To internally validate the models, we used two methods: first, a procedure based on bootstrap resampling as follows: 1,000 bootstrap samples of each imputed dataset including 3,750 patients were drawn with replacement. In each bootstrap resample, the model was fitted and used to calculate Harrell’s C statistic in the bootstrap sample (estimate of apparent performance) and in the original sample (estimate of actual performance). The difference between these two Harrell’s C estimates is an estimate of the optimism bias in the apparent C statistic. In each of the imputed datasets, the optimism was averaged over the 1,000 bootstrap estimates, and the results were pooled over the imputation sets. We also calculated shrinkage slopes using bootstrapping and pooled the slopes over the 10 imputed datasets. As a second method for interval validation, we used 10-fold cross validation, in which the data are randomly split into 10 groups. Prognostic scores are calculated for individuals in each of the 10 groups using a model based on only the individuals of the other nine groups. The prognostics scores are then used to calculate Harrell’s C.

### External validation of prediction models

The Tromsø study is a single-center, population-based prospective cohort study in which repeated health surveys of inhabitants in Tromsø were performed between 1994 and 2016 [31–33]. Between 1994 and 2016, 923 patients were identified with incident VTE, either DVT or PE, and followed up throughout 2016. Patients with planned lifelong anticoagulation (*n* = 73) and those with cancer (*n* = 187) were excluded. Furthermore, an additional 85 patients were excluded from analyses because these patients died or developed recurrent VTE before discontinuation of anticoagulant treatment. Follow-up started at discontinuation of anticoagulant therapy. Patients who had a provoked recurrence during follow-up (*n* = 43) were censored at the recurrence date. Missing values on the FV Leiden mutation (*n* = 133) or ABO blood group (*n* = 59) were imputed, and results were pooled according to Rubin’s rules [29]. Trauma was used as a proxy for cast immobilization in the prediction model, and for a history of cardiovascular disease, only information on myocardial infarction was available. To determine the magnitude of discrimination of the model in the Tromsø data, Harrell’s C-statistic was calculated after pooling results according to Rubin’s rules [29]. Kaplan-Meier plots were created for quintiles of the predicted 2-year recurrence risks. In the Tromsø study, the laboratory markers of models A and B have not been measured, and therefore, only models C and D were externally validated.

All analyses were performed in IBM SPSS Statistics for Windows, version 23.0 (IBM, Armonk, NY, United States); in Stata, version 14 (StataCorp, College Station, TX, USA); and in R, version 3.5.2, using the packages RSM and Survival.

## Results

### Patient characteristics

In total, 3,750 patients from the MEGA follow-up study with a first episode of VTE, without concomitant cancer, were followed for recurrent events for a total of 19,201 person-years. The median duration of follow-up was 5.7 years (IQR 3.2–7.4). Baseline characteristics are shown in Table 1. Mean age of participants was 48 years (SD 13), and 45% were men. Most of the first events were DVT (*n* = 2,231; 59%), and most events were classified as provoked (*n* = 2,592; 69%). Surgery, trauma, and immobilization made up for most of the provoking risk factors.

**Table 1:** Baseline characteristics.

MEGA follow-up study	Patient characteristics
*N*, (%)	3,750 (100%)
Age, mean (SD)	48 (13)
Sex, male (%)	1,684 (45%)
BMI, mean (SD)	27 (4.8)
Type of first event	
DVT	2,231 (59%)
PE	1,184 (32%)
PE + DVT	335 (9%)
Provoked first event*	2,592 (69%)
Trauma, surgery, immobilization†	1,458 (39%)
Prolonged travel	681 (18%)
Pregnancy, puerperium	160 (4%)
Hormone use	1,181 (31%)
Plaster cast	198 (5%)
Unprovoked first event*	1,082 (29%)
Tromsø study	Patient characteristics
*N*, (%)	578 (100%)
Age, mean (SD)	66.3 (14)
Sex, male (%)	279 (48%)
BMI, mean (SD)	27.0 (4.4)
Type of first event	
DVT	348 (60.2%)
PE	194 (33.6%)
PE + DVT	36 (6.2%)
Provoked first event	232 (40%)
Trauma, surgery, immobilization†	190 (33%)
Pregnancy, puerperium	6 (1%)
Hormone use	36 (6%)
Unprovoked first event	346 (60%)

*Some data were missing for some variables (see S1 Table). Because concomitance of provoked risk factors occurred frequently, patients could be counted twice or more.

†Consists of surgery, leg injury, or confinement to bed for more than 3 days at home or in the hospital within 3 months before venous thrombosis.

Abbreviations: DVT, deep vein thrombosis; MEGA, Multiple Environment and Genetic Assessment of Risk Factors for Venous Thrombosis; PE, pulmonary embolism

### Follow-up and outcomes

During follow-up, 507 certain unprovoked recurrent venous thrombotic events were identified, for a total incidence rate of 26.4 per 1,000 person-years (95% CI 24.2–28.8). The cumulative probabilities of unprovoked recurrence after 2, 4, 6, and 8 years were 7.4% (95% CI 6.5%–8.3%), 11.7% (95% CI 10.7%–12.9%), 15.0% (95% CI 13.8%–16.3%), and 17.0% (95% CI 15.6%–18.5%), respectively.

### Predictors

Candidate predictors are provided in S1 Table. Variables presented in Table 2 are those that were included in the final models. In all models, the following clinical parameters were predictive for recurrence: male sex, type and location of first VTE, surgery, pregnancy/puerperium, hormone use, plaster cast, immobility in bed or in hospital, and cardiovascular disease. In model A (maximum model), the following laboratory markers were additionally predictive for recurrence: D-dimer, FVIII antigen, von Willebrand factor, CRP, FV, FX, fibrinogen, and APCsr, whereas none of the genetic factors were predictive. In model B (clinical variables and limited laboratory markers), D-dimer, FVIII antigen, CRP, and FV Leiden were predictive as well, whereas in model C (clinical and genetic variables only), FV Leiden and blood group non-O were also predictive for a recurrent event (Table 2). Of note, because of the predictive nature of the modeling, the coefficients cannot be interpreted causally—i.e., they have no meaning other than indicating an association, conditional on the other included predictors.

**Table 2:** Predictor variables and corresponding regression coefficients included in prediction models A–D.

Type of predictor variables	Model A		Model B		Model C		Model D	
	Coefficient	(95% CI)	Coefficient	(95% CI)	Coefficient	(95% CI)	Coefficient	(95% CI)
**Clinical factors/environmental predictor variables**								
Male sex	0.70	(0.43–0.96)	0.64	(0.38–0.90)	0.63	(0.38;0.89)	0.68	(0.42–0.93)
Type and location of first VTE								
Popliteal^§^ DVT	0.09	(−0.14 to 0.33)	0.11	(−0.12 to −0.34)	0.15	(−0.09 to −0.39)	0.21	(−0.03 to −0.44)
Proximal DVT	0.40	(0.17–0.64)	0.40	(0.16–0.63)	0.46	(0.23–0.70)	0.49	(0.25–0.72)
PE + DVT popliteal^§^ DVT	0.38	(0.06–0.70)	0.41	(0.09–0.73)	0.47	(0.15–0.79)	0.51	(0.20–0.83)
Surgery	−0.42	(−0.78 to −0.05)	−0.40	(−0.76 to −0.03)	−0.51	(−0.88 to −0.15)	−0.52	(−0.88 to −0.16)
Pregnancy/puerperium	−1.31	(−2.3 to −0.30)	−0.51	(−0.83 to −0.18)	−1.49	(−2.50 to −0.48)	−1.44	(−2.45 to −0.43)
Hormone use	−0.59	(−0.92 to −0.26)	−0.70	(−1.28 to −0.12)	−0.67	(−0.99 to −0.35)	−0.62	(−0.95 to −0.30)
Plaster cast	−0.73	(−1.31 to −0.15)	−0.32	(−0.68 to −0.03)	−0.79	(−1.37 to −0.22)	−0.83	(−1.40 to −0.25)
Immobility in bed, in hospital	−0.30	(−0.66 to 0.06)	−0.44	(−0.87 to −0.01)	−0.31	(−0.67 to −0.05)	−0.34	(−0.69 to 0.02)
History of cardiovascular disease	−0.43	(−0.86 to 0.01)	−1.34	(−2.36 to −0.33)	−0.36	(−0.79 to 0.07)	−0.37	(−0.80 to 0.06)
**Genetic factors/genetic predictor variables**								
Blood group, non-O versus O					0.24	(0.03–0.45)		
Factor V Leiden mutation			0.38	(0.16–0.60)	0.40	(0.19–0.61)		
**Laboratory factors/hemorheologic and coagulation predictor variables per unit increase**								
lnDdimer (ng/mL)*	0.26	(0.05–0.47)	0.24	(0.04–0.44)				
lnFactor VIII antigen (IU/dL)*	0.47	(−0.11 to 1.05)	0.81	(0.43–1.19)				
Von Willebrand factor (IU/dL)	0.39	(−0.15 to 0.93)						
lnCRP*			−0.09	(−0.19 to 0.00)				
Factor V (IU/dL)	0.50	(−0.16 to 1.17)						
Factor X (IU/dL)	0.01	(0.00–0.02)						
Fibrinogen (g/L)	−0.24	(−0.42 to 0.05)						
lnAPC ratio*	0.25	(0.10–0.40)						

Model A denotes the maximum model (i.e., all candidate predictor variables); model B, clinical variables and some laboratory markers easy to assess in the clinic; model C, clinical and genetic variables only; model D, clinical variables only. Variables presented in the table were included by a backward selection procedure (entry *p* < 0.15).

*A log-transformation was decided after a nonnormal distribution was found visually.

^§^Indicates DVT at the level of the vena poplitea or below.

Abbreviations: APC, activated protein C; CRP, C-reactive protein; DVT, deep vein thrombosis; PE, pulmonary embolism

### Predictive performance of the model

To determine the magnitude of discrimination of models A–D, Harrell’s C-statistic was calculated (Table 3). The analyses showed that the maximum model had the highest predictive capability, with a C-statistic of 0.73 (95% CI 0.71–0.76). The discriminative performance was somewhat lower in the other models, with C-statistics of 0.72 (95% CI 0.70–0.75) for model B, 0.70 (95% CI 0.68–0.73) for model C, and 0.69 (95% CI 0.67–0.72) for model D. When type of first event (provoked versus unprovoked) was the only variable used (i.e., the current clinical situation), the C-statistic was substantially lower, at 0.61 (95% CI 0.58–0.63). A sensitivity analysis using the complete data only (i.e., in the nonimputed data) yielded very similar results for the Harrell’s C-statistics (Table 3).

**Table 3:** Predictive performance of prediction models A–D.

Predictive performance	Model A		Model B		Model C		Model D	
Harrell’s C	0.73	(0.71–0.76)	0.72	(0.70–0.75)	0.70	(0.68–0.73)	0.69	(0.67–0.72)
Harrell’s C corrected for optimism by bootstrap	0.73		0.72		0.70		0.70	
Harrell’s C after 10-fold cross validation	0.72	(0.69–0.74)	0.71	(0.69–0.73)	0.69	(0.67–0.72)	0.69	(0.66–0.71)
Harrell’s C after censoring at 2 years of follow-up	0.73	(0.70–0.77)	0.72	(0.69–0.75)	0.70	(0.67–0.73)	0.69	(0.66–0.72)
Harrell’s C using complete cases only	0.74	(0.70–0.77)	0.73	(0.69–0.76)	0.71	(0.68–0.73)	0.69	(0.67–0.71)
Range of 2-year predicted risks*	0%–54%		0%–46%		0%–32%		0%–24%	
Harrell’s C after external validation	NA		NA		0.64	(0.62–0.66)	0.65	(0.63–0.66)
Baseline 2-year recurrence-free probability*	.9998462		.9996196		.9235595		.9019939	

Model A denotes maximum model (i.e., all candidate predictor variables); model B, clinical variables and some laboratory markers easy to assess in the clinic; model C, clinical and genetic variables only; model D, clinical variables only.

*The baseline recurrence-free probability S_0_ can be used to calculate the absolute 2-year risk of recurrence for a patient using the following equation: risk of recurrence = 1 − S_0_** exp(prognostic score). Here, the prognostic score is equal to beta1*x1 + beta2*x2 + beta3*x3 + …, where the x1, x2 x3, etc., represent the variables in the prediction model, and beta1, beta2, beta3, etc., represent the corresponding regression coefficients.

Abbreviation: NA, not applicable

**Fig 2:**
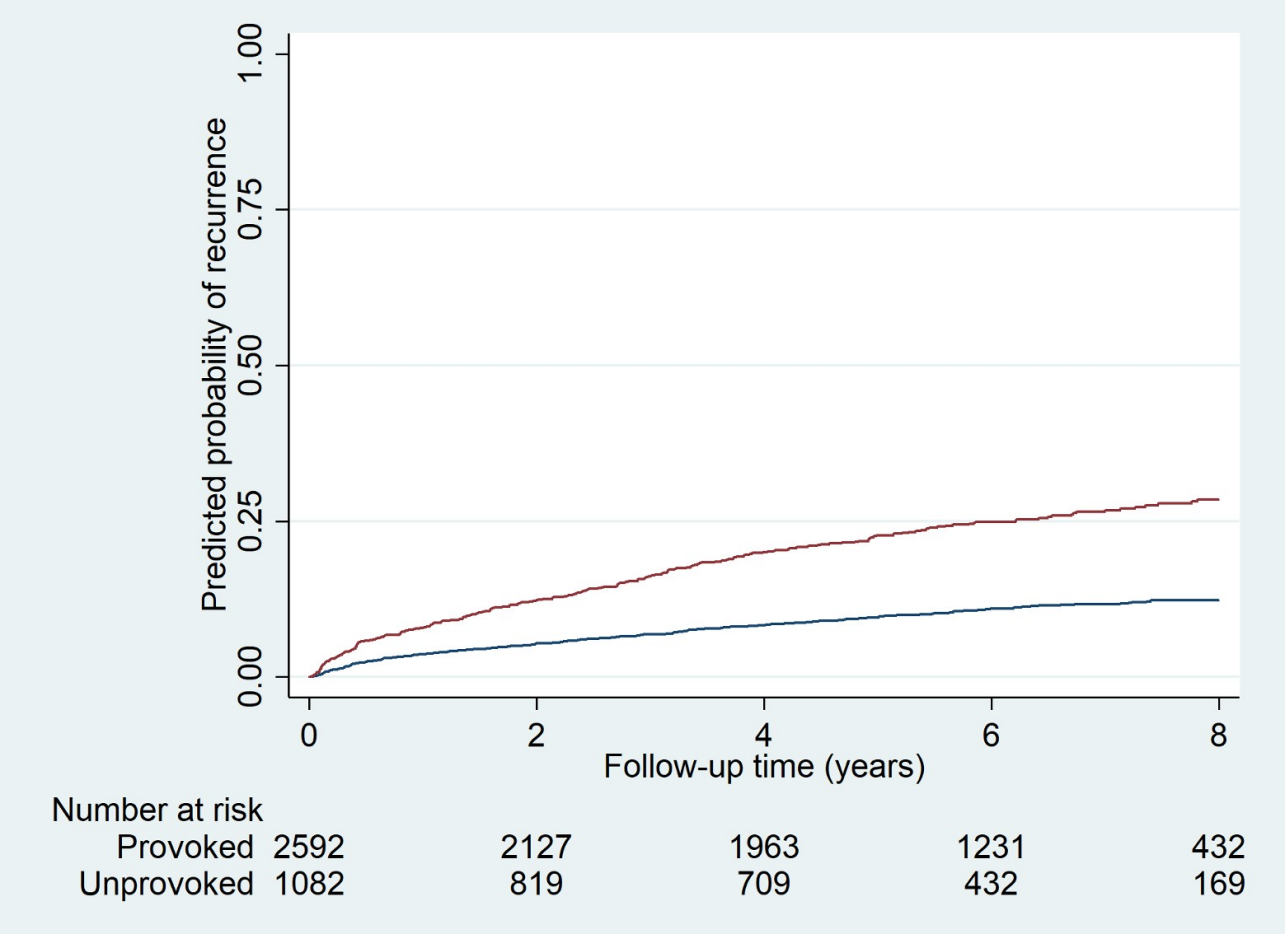
Probability of recurrence in patients with first provoked and first unprovoked VTE. VTE, venous thromboembolism.

**Fig 3:**
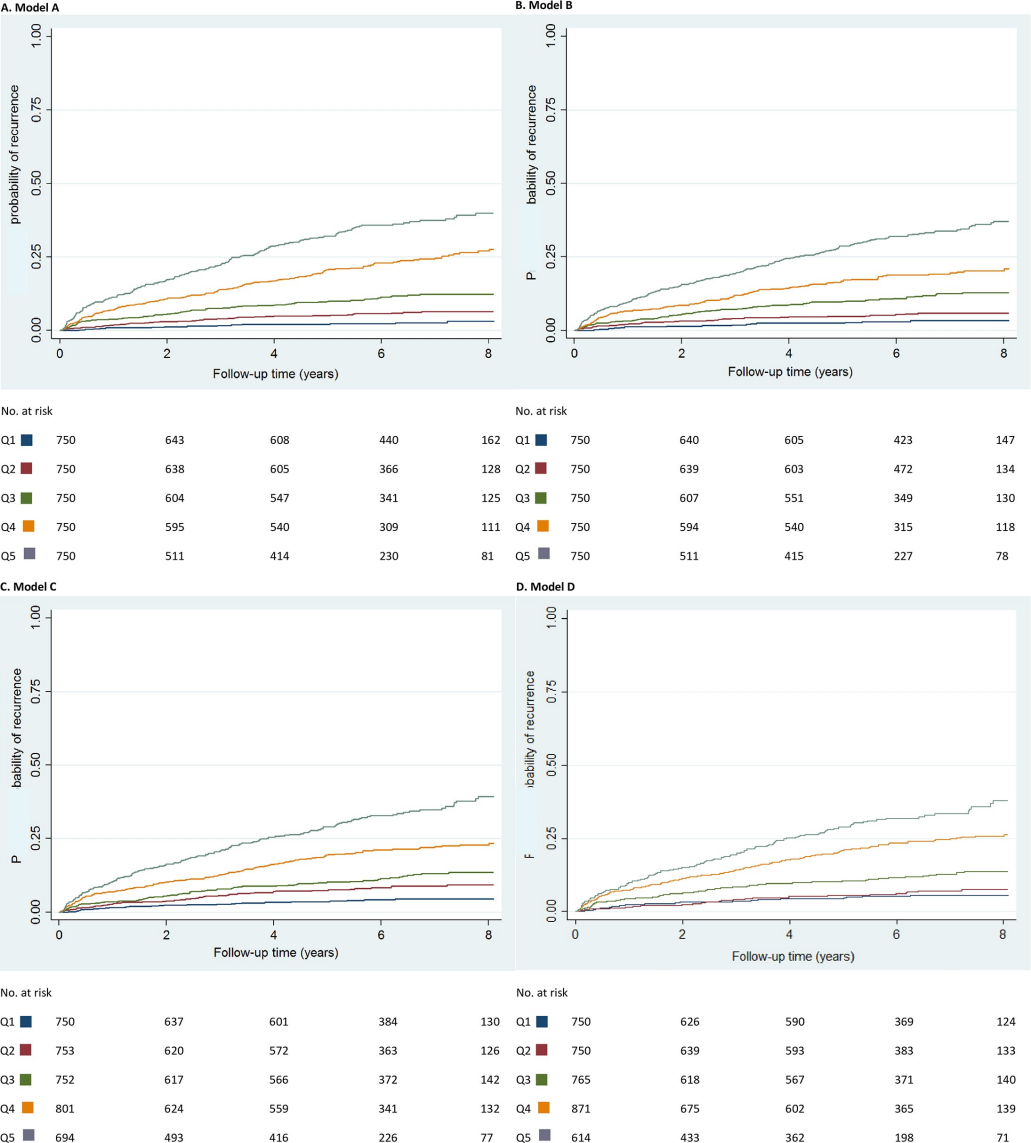
(A–D) Probability of recurrence stratified by Qs of prognostic index. No., number; Q, quintile.

Figs 2 and 3 show Kaplan-Meier curves for the current clinical situation (i.e., displaying the actual observed risks) and for models A–D, according to quintiles of the prognostic score, respectively. Increasing quintiles of the prognostic score corresponded in a dose-response manner to increased observed risks of recurrence. The extent to which the five risk groups could be distinguished increased with the number of variables included in the model (i.e., highest for model A [Fig 3A] and lowest for the current clinical situation [Fig 2]). Likewise, inclusion of more variables led to a wider range of risks that the models could detect, which was largest for model A and smallest for model D (0%–54% for model A and 0%–24% for model D [Table 3]).

**Fig 4:**
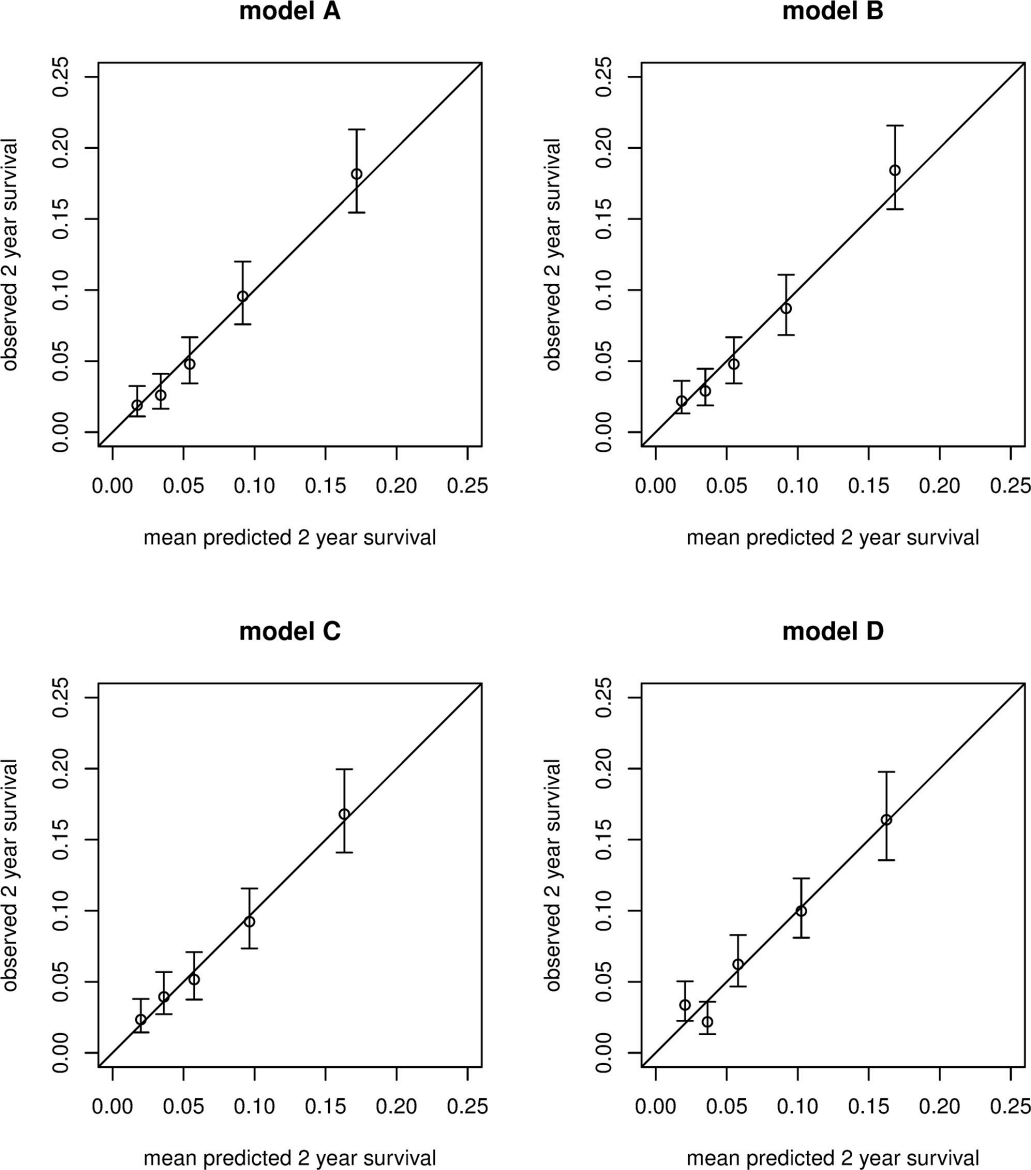
Calibration of observed 2-year recurrence risks plotted against the estimated predicted recurrence risks for the four models.

Fig 4 shows the calibration plots in which the 2-year observed risks, from a Kaplan-Meier calculation in five groups, split on the basis of their xbetas, are plotted against the mean 2-year risks as estimated by the four models. The plots show excellent calibration for all models, as expected, because calibration is performed here in the same dataset in which the model was developed.

### Internal and external validation

Two internal validation procedures showed that the degree of optimism bias of the models was minimal (Table 3). The shrinkage slopes for the models were close to 1: 0.950 for model A, 0.959 for model B, 0.953 for model C, and 0.960 for model D.

**Fig 5:**
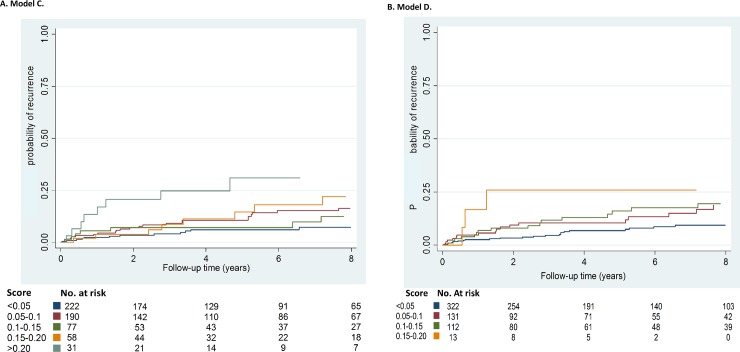
Probability of recurrence stratified by categories of prognostic index in the external validation cohort for models C (A) and D (B). No., number.

**Fig 6:**
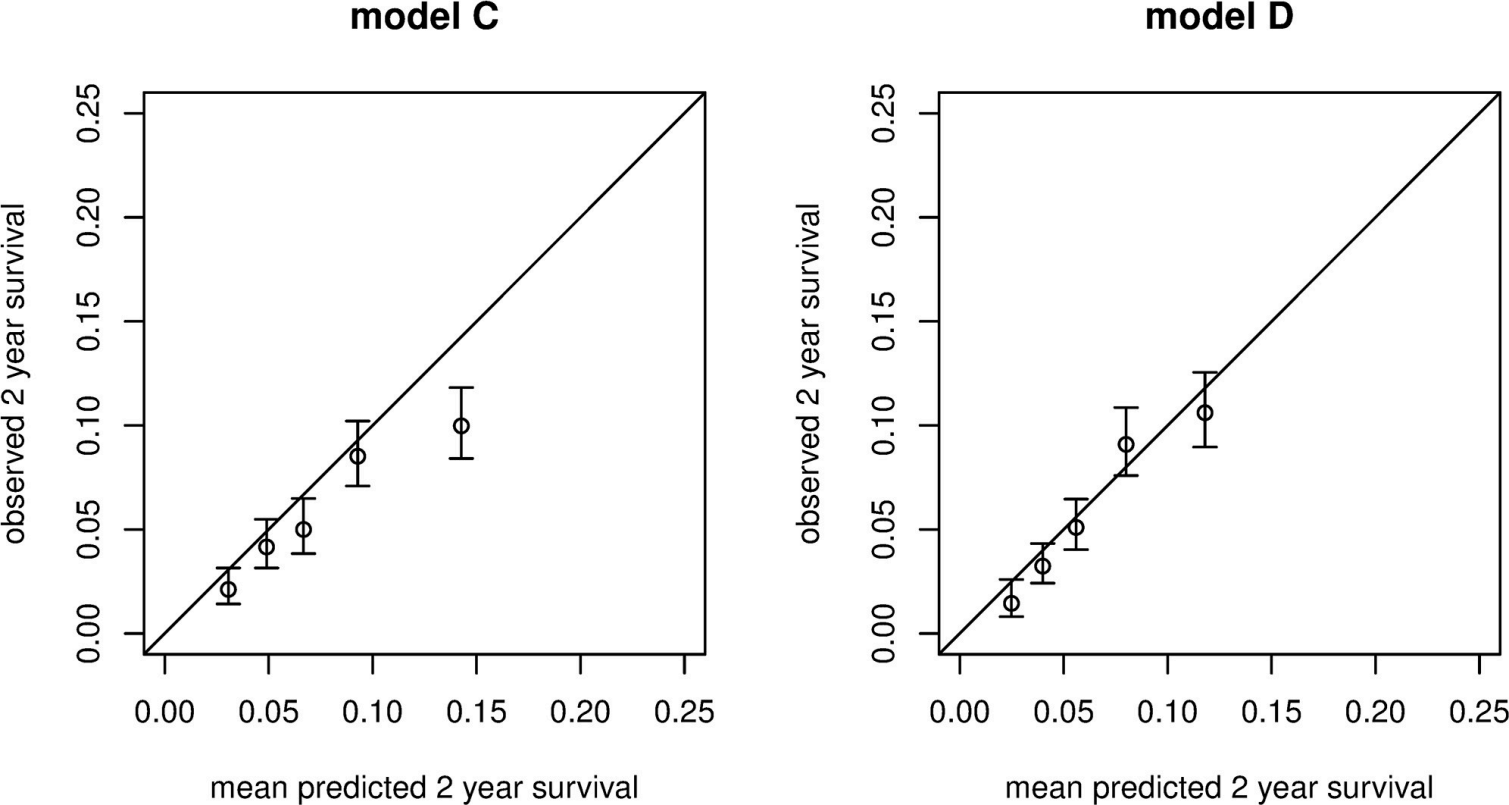
Calibration of observed 2-year recurrence risks plotted against the estimated predicted recurrence risks for models C and D in the validation cohort.

In the Tromsø study, 73 out of 587 patients developed an unprovoked recurrence during a median follow-up time of 5.0 years, which corresponded to a recurrence rate of 20.1 (95% CI 16.0–25.3) per 1,000 person-years. Including only type of first event (provoked versus unprovoked)—i.e., the current clinical situation—led to a C-statistic of 0.57 (95% CI 0.55–0.59). Models C and D were externally validated, and the Harrell’s C-statistics were 0.64 (95% CI 0.62–0.66) and 0.65 (95% CI 0.63–0.66), respectively. The corresponding Kaplan-Meier curves are shown in Fig 5, and the calibration of models C and D is shown in Fig 6. The predicted 2-year recurrence risks for the five risk groups were very close to the observed 2-year risks, which indicates that the models performed well and calibration was good.

### Absolute 2-year predicted risks

**Fig 7:**
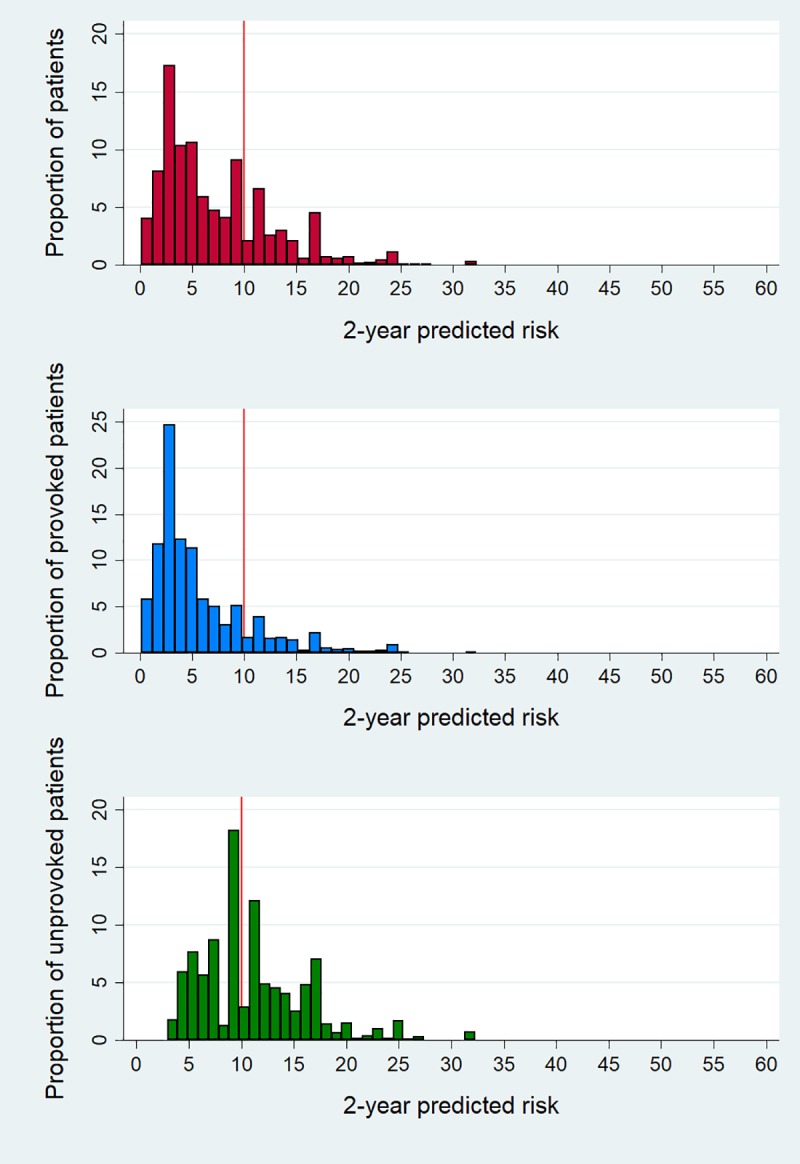
Histogram of 2-year predicted risks according to the maximum model in total population (top), patients with a provoked first venous thromboembolism (middle), and patients with an unprovoked first venous thromboembolism (bottom).

Fig 7A–7C depicts the distribution of individual 2-year predicted risks of recurrent VTE according to the maximum model (model A). As shown in Fig 7A, 989 out of 3,750 (26%) patients had a predicted risk of recurrent VTE of >10% at 2 years of follow-up. For patients with provoked first events (*n* = 2,592), there were 367 (15%) patients who had a predicted risk of recurrence of >10% (Fig 7B). In patients whose first event was unprovoked, 484 (45%) had a predicted risk of recurrence that was less than 10% (Fig 7C). Hence, 23% (367 + 484/3,750) of the total cohort was misclassified, of whom 367 patients would have been undertreated and 484 patients would have been overtreated if the guidelines had been strictly followed (without allowing for bleeding risk or patient preferences). For the other models, with slightly lower discriminative performances, these misclassification proportions would be somewhat lower (see S1 Fig on model C).

## Discussion

One of the most important challenges in the management of VTE is how to deal with its considerable recurrence risk [1–4]. Prolonged anticoagulant treatment offers effective prevention but is not universally applicable because of its associated bleeding risk [3,27]. As both risks will persist over a person’s lifetime, the cumulative probability of either a thrombotic or a bleeding event is high [6,7]. The decision of continuation or discontinuation of treatment should therefore be informed [3,9].

Currently, guidelines distinguish two risk groups—i.e., patients with unprovoked or provoked first events—and generally advise the former to continue and the latter to stop anticoagulation [3,27]. Problems with this approach are that classifying a patient as having had an unprovoked event is not clear-cut [11] and that the risk varies within these groups [13]; hence, patients with a provoked event and a considerable recurrence risk can be withheld treatment, and patients with an unprovoked event but low recurrence risk are unnecessarily treated and exposed to a bleeding risk. Some prediction models have been developed in an attempt to improve this situation and to refine risk stratification, the three best known of which are the “men continue and HERDOO2” rule, the Vienna prediction model, and the DASH score [15–17,34–37]. However, limitations of the existing models are that (1) they only apply to patients with an unprovoked first event; (2) they included a limited number of predictors, which simplifies their use in practice but limits their discriminative performance; and (3) they included provoked recurrent events in the derivation phase, in which events are inherently unpredictable. We developed four versions of a new model—the Leiden Thrombosis Recurrence Risk Prediction (L-TRRiP) model, which has as its advantages that it applies to all patients with first thrombosis and was derived from a comprehensive set of variables—with the aim to predict unprovoked recurrent thrombosis. The model showed good discriminative performance, with a wide range of predicted absolute risks, showing that much can be gained by further refined risk stratification compared with the current situation (C-statistic of 0.61; only two risk categories possible [Fig 2]).

### Strengths and limitations

To the best of our knowledge, the MEGA follow-up study is the largest population-based study on recurrent venous thrombosis to date, including 3,750 patients, with 507 recurrent events and >100 clinical, genetic, and biomarker variables. Its size allowed inclusion of a large number of candidate predictor variables [39,40]. With a rule of thumb of no fewer than 10 events per variable (EPV), we could include 39 predictor variables (47 parameters) for maximum model A, and the number was lower (and hence the EPV higher) for models B–D, implying that they were unlikely to have been overfitted. In contrast, the HERDOO2 model was markedly overfitted, with collected information on 69 predictors, of which 36 were considered, whereas there were only 91 recurrent events (EPV ≤ 2.5) [10,15]. Another strength of our study is that we, similar to HERDOO2, Vienna, and DASH, collected data using a prospective design, which limits detection bias and ensures uniformity of definitions [15–17]. Furthermore, also similar to the Vienna model, but not to HERDOO2 and DASH [15–17], we avoided dichotomous categorization of continuous candidate predictors, which is suboptimal, because informative variation of values within each class is not used [38]. Another strength is that we used a Cox proportional hazards model, which takes into account the censoring of patients over time and the variable lengths of individual follow-up. Model performance was evaluated by using internal validation methods that corrected for optimism bias and by external validation with use of follow-up data from the Tromsø study (73 events out of 587 patients), which showed good results both in terms of discrimination and calibration. To our knowledge, the Tromsø study is the only other cohort in the world in which detailed data are available on an unselected group of patients with first VTE.

Some limitations of the present study also need to be addressed. First, coagulation factor levels were measured only once during follow-up (i.e., at baseline). However, serial measurements of coagulation factors over time have shown that they remain reasonably constant [41,42]. Second, coagulation factor levels were not measured in all patients, because blood sampling for the study was discontinued halfway during the study for logistic reasons. Other data were also occasionally not complete, but all missing data were imputed. That bias due to the imputation is unlikely is reflected by the results of the complete case analysis, in which the Harrell’s C-statistics were found to be very similar. Third, because we used a strict definition of unprovoked recurrent VTE and included only certain unprovoked recurrences in our study, incidence rates and cumulative incidences may have been slightly underestimated. However, using a strict and well-defined outcome as we did will have decreased misclassification and hence improved discriminatory performance. Fourth, our study population consisted of 90% (Northern) European descent, and we excluded children and individuals aged >70 years at the time of the first event. Therefore, results from MEGA may not be generalizable to other ethnic groups, and our findings cannot be extrapolated to children or the elderly. Limitations of the Tromsø study are that the number of recurrences was relatively low, which leaves some imprecision around the estimates of the C-statistics. Furthermore, information on FV Leiden and blood group was missing in almost one-third of the patients and needed to be imputed, and not all clinical information was present as defined in the MEGA data, so proxies needed to be used. Also, the laboratory markers that we used for models A and B were not available here, so these models could not be externally validated. This may have limited the external validation of the models. Lastly, we excluded patients with a history of cancer because we could not always be 100% certain that these patients had not had a relapse of their cancer. However, exclusion of these patients would not have affected our study in any other way than having a better-defined patient population and a small loss of power. Still, it needs to be realized that the model does not apply to patients with a history of cancer.

### Choice of model

In the important decision on the duration of anticoagulant treatment, with life-long implications, it is worthwhile to include as many variables as possible for the greatest discriminatory power. At the same time, the model should be feasible in clinical practice, which again limits the number of predictors. We developed four versions of the L-TRRiP model, which allows a choice by balancing maximum discriminative performance (model A: maximum number of predictors) versus optimal ease of use (model D: clinical variables only). Model A shows the best performance, not only in terms of the C-statistic but also, more important clinically, in the wide range of absolute risks that it can distinguish (2-year predicted risks between 0% and 54%). These performance markers are lowest for model D. However, model A requires extensive laboratory testing (7 markers included) for which interruption of anticoagulant treatment is warranted for correct interpretation of the values. This is less feasible for the clinic. At the other end of the spectrum is model D, which requires no laboratory measurements but has a lower C-statistic and half the size of the range of risks that can be detected (0%–24%). We would therefore propose to choose model C, which has a slightly higher C-statistic, but its added value over model D is mostly that its range of detected risks is much wider (0%–32%). Besides, this model has been externally validated (in contrast to models A and B), in which it performed reasonably well. For model C, only two laboratory markers are required—i.e., blood type and FV Leiden. Obtaining these is easy (they can be measured almost universally, even from a buccal swab), and most importantly, interruption of anticoagulant treatment is not necessary, because this will obviously not influence these genetic markers.

### Potential clinical implications

We showed that the 2-year predicted risks of all patients vary strongly according to our model (Fig 7) and also within the groups that are now considered low (provoked) or high (unprovoked) risk. Of patients with a provoked first event (who now all stop treatment), 15% had a predicted risk of >10% of recurrence at 2 years of follow-up, which would be considered sufficiently high to warrant prolonged anticoagulant treatment [30]. In contrast, we observed that in patients with a first unprovoked event (who now mostly go on with treatment), 45% had an estimated risk of recurrence of <10% at 2 years of follow-up and could therefore consider stopping anticoagulant treatment. Hence, if the guidelines had been applied to this cohort, based on this model, 23% of all patients were misclassified and under- or overtreated, consequently leading to preventable recurrent VTE and bleeds, respectively. The poor performance of using only (un)provoked VTE as a determinant for treatment is also reflected in the low C-statistic for the current situation, at 0.61.

Compared with existing models that predict recurrent VTE [15–17], our L-TRRiP model has as its most important clinical advantage that it can be applied to all patients with a first thrombosis, so it is not necessary to determine first whether a patient fulfills the criteria for a particular model. Furthermore, a patient does not need to discontinue anticoagulation before assessing his/her recurrence risk. Our model may form a basis for optimal individualized treatment, in which a patient’s bleeding risk should also be taken into account. This is currently possible with a model such as the VTE-BLEED [43,44]. Obviously, the exact form of such personalized treatment should be tested in a management study, in which treatment based on an individual’s L-TRRiP and VTE-BLEED score is compared with routine treatment according to the current guidelines. For easy use of the L-TRRiP score in such a study or in the clinic, we will develop a web- or mobile device–based app, using the algorithm developed in model C. To the same app, an algorithm for a bleeding risk model can be added. Such an application could even be incorporated in an electronic patient file, in which the estimated risks will automatically appear when all data for the algorithm are available.

## Conclusion

The current guidelines and the available prediction models fall short in providing optimal care for patients with a first VTE, which leads to preventable recurrent thrombosis and bleeding. The model we propose has important advantages because it applies to patients with provoked or unprovoked first VTE—except for patients with (a history of) cancer—allows a refined risk stratification, and is still easily usable in clinical practice. Personalized anticoagulant treatment, based on this model and on a model that assesses bleeding risk, should be further explored in a management study.

## Supporting information

S1 Table Candidate predictor variables. Multiple imputation was used to complete missing predictor values, of which the list below gives an overview. Data on clinical factors were collected by means of a questionnaire; missing data on the questionnaire resulted in missing data reported in the table. Blood collection was terminated for logistic reasons on May 31, 2002. For participants included after this date, no blood was sampled, and buccal swabs were collected for DNA analyses. Patients who did not return their buccal swab had missing data for the DNA variables.
(DOCX)

S1 Fig Histogram of 2-year predicted risks according to model C: (A) total population; (B) patients with a provoked first VTE, and (C) patients with an unprovoked first VTE. VTE, venous thromboembolism.
(TIF)

S1 Text Supplementary methods. Classification and definition of recurrent events and laboratory analyses.
(DOCX)

S1 Protocol MEGA protocol. MEGA, Multiple Environment and Genetic Assessment of Risk Factors for Venous Thrombosis.
(PDF)

## Abbreviations

APC: activated protein C
APCsr: activated protein C resistance
CRP: C-reactive protein
DASH: D-dimer, Age, Sex, Hormonal therapy
DVT: deep vein thrombosis
EPV: events per variable
FV: factor V
FVIII: factor VIII
FX: factor X
ISTH: International Society on Thrombosis and Haemostasis
L-TRRiP: Leiden Thrombosis Recurrence Risk Prediction
MEGA: Multiple Environment and Genetic Assessment of Risk Factors for Venous Thrombosis
NA: not applicable
PE: pulmonary embolism
SNP: single nucleotide polymorphism
VTE: venous thromboembolism
