# Correction: Prediction of recurrent venous thrombosis in all patients with a first venous thrombotic event: The Leiden Thrombosis Recurrence Risk Prediction model (L-TRRiP)

**DOI:** 10.1371/journal.pmed.1003612

**Published:** 2021-04-23

**Authors:** Jasmijn F. Timp, Sigrid K. Braekkan, Willem M. Lijfering, Astrid van Hylckama Vlieg, John-Bjarne Hansen, Frits R. Rosendaal, Saskia le Cessie, Suzanne C. Cannegieter

In Table 2, the coefficients of the prediction model are incorrect for rows 3–5 of the clinical factors/environmental predictor variables, and for some of the coefficients of model B). The correct table is below.

**Table 2:** Predictor variables, and corresponding regression coefficients, included in prediction models A-D.

	*Model A*		*Model B*		*Model C*		*Model D*	
	Coefficient	(95%CI)	Coefficient	(95%CI)	Coefficient	(95%CI)	Coefficient	(95%CI)
*Clinical factors/ Environmental predictor variables*								
Male sex	0.70	(0.43;0.96)	0.64	(0.38;0.90)	0.63	(0.38;0.89)	0.68	(0.42;0.93)
Type of 1^st^ VT (DVT, PE, PE+DVT) PE PE+DVT	-0.50 0.28	(-0.76;-0.24) (0.02;0.55)	-0.50 0.30	(-0.76;-0.25) (0.04;0.57)	-0.61 0.32	(-0.87;-0.36) (0.06;0.58)	-0.69 0.31	(-0.95;-0.44) (0.04;0.57)
Location of DVT (PE, distal DVT, proximal DVT) Popliteal§ and distal VT Proximal DVT	-0.40 0	(-0.63;-0.17) omitted	-0.39 0	(-0.63;-0.16) omitted	-0.46 0	(-0.69;-0.23) omitted	-0.49 0	(-0.72;-0.25) omitted
Surgery	-0.42	(-0.78;-0.05)	-0.40	(-0.76;-0.03)	-0.51	(-0.88;-0.15)	-0.52	(-0.88;-0.16)
Pregnancy/ puerperium	-1.31	(-2.3;-0.30)	-1.34	(-2.36;-0.33)	-1.49	(-2.50;-0.48)	-1.44	(-2.45;-0.43)
Hormone use	-0.59	(-0.92;-0.26)	-0.51	(-0.83;-0.18)	-0.67	(-0.99;-0.35)	-0.62	(-0.95;-0.30)
Plaster cast	-0.73	(-1.31;-0.15)	-0.70	(-1.28;-0.12)	-0.79	(-1.37;-0.22)	-0.83	(-1.40;-0.25)
Immobility in bed, in hospital	-0.30	(-0.66;0.06)	-0.32	(-0.68;-0.03)	-0.31	(-0.67;-0.05)	-0.34	(-0.69;0.02)
History of cardiovascular disease	-0.43	(-0.86;0.01)	-0.44	(-0.87;-0.01)	-0.36	(-0.79;0.07)	-0.37	(-0.80;0.06)
*Genetic factors/ Genetic predictor variables*								
Blood group, non-O versus O					0.24	(0.03;0.45)		
Factor V Leiden mutation			0.38	(0.16–0.60)	0.40	(0.19;0.61)		
*Laboratory factors/ Hemorheologic and coagulation predictor variables per unit increase*								
lnDdimer (ng/mL)*	0.26	(0.05;0.47)	0.24	(0.04;0.44)				
lnFactor VIII antigen (IU/dL)*	0.47	(-0.11;1.05)	0.81	(0.43;1.19)				
Von Willebrand factor (IU/dL)	0.39	(-0.15;0.93)						
lnCRP*			-0.09	(-0.19;0.00)				
Factor V (IU/dL)	0.50	(-0.16;1.17)						
Factor X (IU/dL)	0.01	(0.00;0.02)						
Fibrinogen (g/L)	-0.24	(-0.42;0.05)						
lnAPC ratio*	0.25	(0.10;0.40)						

Model A denotes the maximum model (i.e., all candidate predictor variables); model B, clinical variables and some laboratory markers easy to assess in the clinic; model C, clinical and genetic variables only; model D, clinical variables only. Variables presented in the table were included by a backward selection procedure (entry p < 0.15).

*A log-transformation was decided after a nonnormal distribution was found visually.

§ Indicates DVT at the level of the vena poplitea or below.

Abbreviations: APC, activated protein C; CRP, C-reactive protein; DVT, deep vein thrombosis; PE, pulmonary embolism
