# Supplementary material for: Prediction of recurrent venous thrombosis in all patients with a first venous thrombotic event: The Leiden Thrombosis Recurrence Risk Prediction model (L-TRRiP)
Source: PLoS Med. 2019 Oct 11;16(10):e1002883. doi: 10.1371/journal.pmed.1002883 (PMC6788686; doi:10.1371/journal.pmed.1002883)
Supplement: S1 Table — Multiple imputation was used to complete missing predictor values, of which the list below gives an overview. Data on clinical factors were collected by means of a questionnaire; missing data on the questionnaire resulted in missing data reported in the table. Blood collection was terminated for logistic reasons on May 31, 2002. For participants included after this date, no blood was sampled, and buccal swabs were collected for DNA analyses. Patients who did not return their buccal swab had missing data for the DNA variables. (DOCX) [file pmed.1002883.s001.docx]

| **S1 Table. Candidate predictor variables** | | | | | | | | | | | |
| --- | --- | --- | --- | --- | --- | --- | --- | --- | --- | --- | --- |
|  |  |  |  |  |  |  |  |  |  |  |  |
|  |  |  |  | Candidate predictor in model: | | | | Included predictor in model: | | | |
| *Clinical factors/ Environmental predictor variables* | |  | *Missing, N (%)* | *A* | *B* | *C* | *D* | *A* | *B* | *C* | *D* |
| Age | Continuous |  | 0 (0%) | X | X | X | X |  |  |  |  |
| Sex | Categorical |  | 0 (0%) | X | X | X | X | X | X | X | X |
| Type of 1^st^ VT (DVT, PE, PE+DVT) | Categorical |  | 0 (0%) | X | X | X | X | X | X | X | X |
| Proximal vs popliteal^§^ DVT | Categorical |  | 880 (23%) | X | X | X | X | X | X | X | X |
| Postthrombotic syndrome, mild or severe | Categorical | Based on Villalta score assessed at moment of blood collection | 1110 (30%) | X | X | X | X |  |  |  |  |
| BMI | Continuous |  | 304 (8%) | X | X | X | X |  |  |  |  |
| Surgery | Categorical | Within 3 months before VT | 13 (0%) | X | X | X | X | X | X | X | X |
| Provoked, additional factors | Categorical | Including: confinement to bed >=3 days at home, pneumonia in year before VT, other infection in year before VT (urinary tract infection, pyelonephritis, arthritis, bursitis, sinusitis, pulpitis, inflammation elsewhere, hepatitis A, B or C), leg injury in 3 months before VT, prolonged travel >4 hours within 2 months before VT | 270 (7%) | X | X | X | X |  |  |  |  |
| Pregnancy/ puerperium | Categorical | Within 3 months before VT | 16 (0%) | X | X | X | X | X | X | X | X |
| Hormone use | Categorical | At the time of VT, including: hormone replacement therapy and hormonal contraceptives | 41 (1%) | X | X | X | X | X | X | X | X |
| Plaster cast | Categorical | Within 3 months before VT | 0 (0%) | X | X | X | X | X | X | X | X |
| Immobility in bed, in hospital | Categorical | Confinement to bed >=3 days in hospital, within 3 months before VT | 0 (0%) | X | X | X | X | X | X | X | X |
| Cardiovascular disease | Categorical | Including a history of: heart failure, angina pectoris, arterial insufficiency of legs (claudication), acute MI | 227 (6%) | X | X | X | X | X | X | X | X |
| Cerebrovascular disease | Categorical | Including a history of: ischemic stroke, transient ischemic attack, intracerebral haemorrhage | 227 (6%) | X | X | X | X |  |  |  |  |
| Disease, additional comorbidities | Categorical | Including a history of (or currently present): diabetes type I or II, liver Xdisease, kidney disease, rheumatoid arthritis, bronchitis, emphysema, hyper- and hypothyroidism | 227 (6%) | X | X | X | X |  |  |  |  |
|  |  |  |  |  |  |  |  |  |  |  |  |
| *Genetic factors/ Genetic predictor variables* | |  |  |  |  |  |  |  |  |  |  |
| Blood group, non-O vs O | Categorical |  | 329 (9%) | X | X | X |  |  |  | X |  |
| Factor V Leiden mutation | Categorical | Homozygous or heterozygous (vs 1691GG) | 314 (8%) | X | X | X |  |  | X | X |  |
| SNPscore | Categorical | Sum of SNPs risk allele present (homozygous or heterozygous): factor V Leiden mutation, prothrombin mutation, SNPrs8176719, SNPrs2066865, SNPrs2036914 | 2346 (63%) | X | X | X |  |  |  |  |  |
|  |  |  |  |  |  |  |  |  |  |  |  |
| *Laboratory factors/ Hemorheologic and coagulation predictor variables* | |  |  |  |  |  |  |  |  |  |  |
| Ddimer (ng/mL) | Continuous | Log-transformed* | 1834 (49%) | X | X |  |  | X | X |  |  |
| Factor VIII antigen (IU/dL) | Continuous | Log-transformed* | FVIIIag: 1645 (44%) | X | X |  |  | X | X |  |  |
| Von Willebrand factor (IU/dL) | Continuous | Log-transformed* | 1643 (44%) | X | X |  |  | X |  |  |  |
| Hemoglobin | Continuous |  | 1669 (45%) | X | X |  |  |  |  |  |  |
| White Blood cell Count | Continuous | Log-transformed* | 1669 (45%) | X | X |  |  |  |  |  |  |
| Monocyte percentage | Continuous | Log-transformed* | 1690 (45%) | X | X |  |  |  |  |  |  |
| Red cell Distribution Width | Continuous | Log-transformed* | 1670 (45%) | X | X |  |  |  |  |  |  |
| CRP | Continuous | Log-transformed* | 1644 (44%) | X | X |  |  |  | X |  |  |
| Factor VII (IU/dL) | Continuous |  | 1829 (49%) | X |  |  |  |  |  |  |  |
| Factor V (IU/dL) | Continuous |  | 1643 (44%) | X |  |  |  | X |  |  |  |
| Factor X (IU/dL) | Continuous |  | 1829 (49%) | X |  |  |  | X |  |  |  |
| Protein S, free (IU/dL) | Continuous | Log-transformed* | 1839 (49%) | X |  |  |  |  |  |  |  |
| Protein C (IU/dL) | Continuous |  | 1829 (49%) | X |  |  |  |  |  |  |  |
| Fibrinogen (g/L) | Continuous |  | 1643 (44%) | X |  |  |  | X |  |  |  |
| Factor II (IU/dL) | Continuous |  | 1829 (49%) | X |  |  |  |  |  |  |  |
| Factor IX (IU/dL) | Continuous |  | 1830 (49%) | X |  |  |  |  |  |  |  |
| Factor XI (IU/dL) | Continuous |  | 1643 (44%) | X |  |  |  |  |  |  |  |
| TFPI (U/mL) | Continuous |  | 1649 (44%) | X |  |  |  |  |  |  |  |
| Antithrombin (IU/dL) | Continuous |  | 1643 (44%) | X |  |  |  |  |  |  |  |
| APC ratio | Continuous | Log-transformed* | 1772 (47%) | X |  |  |  | X |  |  |  |
| ETP | Continuous |  | 1837 (49%) | X |  |  |  |  |  |  |  |
| *A log-transformation was decided after a non-normal distribution was found visually. ^§^ Indicates DVT at the level of the v.poplitea or below | | | | | | | | | | | |
