# Supplementary figures and images for: Prediction of recurrent venous thrombosis in all patients with a first venous thrombotic event: The Leiden Thrombosis Recurrence Risk Prediction model (L-TRRiP)
Source: PLoS Med. 2019 Oct 11;16(10):e1002883. doi: 10.1371/journal.pmed.1002883 (PMC6788686; doi:10.1371/journal.pmed.1002883)

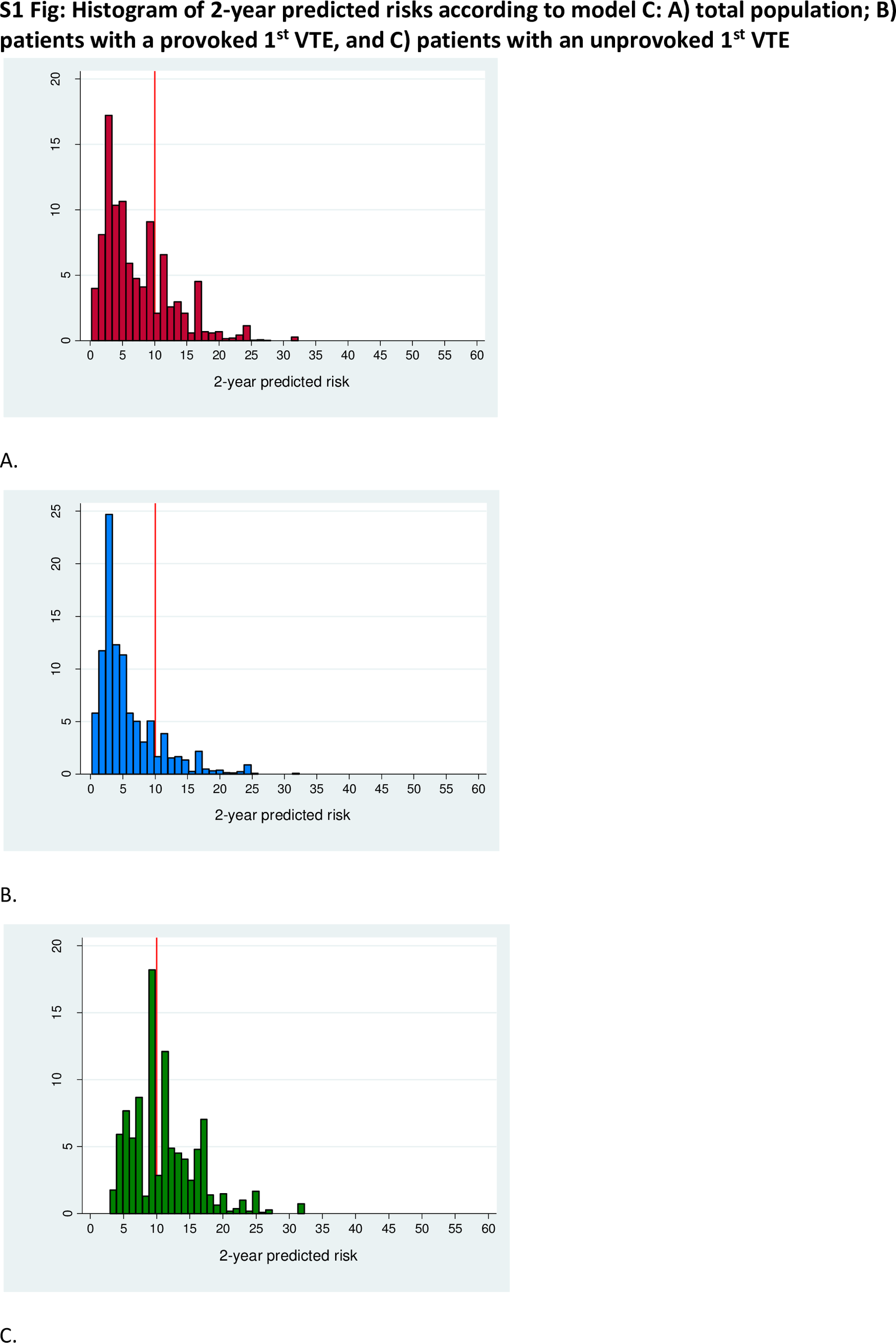

Supplement: S1 Fig — Histogram of 2-year predicted risks according to model C: (A) total population; (B) patients with a provoked first VTE, and (C) patients with an unprovoked first VTE. VTE, venous thromboembolism. (TIF) [file pmed.1002883.s002.tif]
