# Supplementary material for: Prediction of recurrent venous thrombosis in all patients with a first venous thrombotic event: The Leiden Thrombosis Recurrence Risk Prediction model (L-TRRiP)
Source: PLoS Med. 2019 Oct 11;16(10):e1002883. doi: 10.1371/journal.pmed.1002883 (PMC6788686; doi:10.1371/journal.pmed.1002883)
Supplement: S1 Text — Classification and definition of recurrent events and laboratory analyses. (DOCX) [file pmed.1002883.s003.docx]

**S1 Text**

**Supplementary Methods**

**Classification and definition of recurrent events**

To be classified as a certain recurrence, a reported recurrence should fulfil one of the following criteria.

1. A discharge letter was present concluding a diagnosis of recurrence, based on available clinical and radiological data. This recurrence should be in a different vein or in a different part of the body than the first event. The discharge letter had to contain information about instrumental diagnostic procedures. If location of either first or second thrombosis was not known or was similar to the first event, an event was still classified as certain if at least three months had passed since the first thrombosis.
2. A discharge letter was not available (e.g. when treating physician was unknown) but both the anticoagulation clinic and the patient reported a recurrence at a clearly different location than the first event (contralateral leg, DVT after PE or vice versa) or a time period of more than a year had passed between the two events.
3. A registered cause of death from PE or DVT at least six months after the first event.

Uncertain recurrences were defined by four criteria, one of which had to apply:

1. A diagnosis of a possible recurrence in the discharge letter, where clinical and radiological data could not distinguish between an extension of the first and a new thrombotic event.
2. A discharge letter was not available but both the patient and the anticoagulation clinic reported a recurrence within a year after the first event.
3. Information was only available from either the patient or the anticoagulation clinic.
4. A registered cause of death from PE or DVT within six months after the first event.

**Laboratory analyses**

All assays were performed in automated machines by laboratory technicians. Prothrombin (factor II) activity, factor VII activity, factor X activity and factor XI activity were measured with a mechanical clot detection method on a STA-R coagulation analyser following the instructions of the manufacturer (Diagnostica Stago, Asnieres, France). Levels of factor IX antigen, factor VIII antigen and factor V antigen, were determined by enzyme-linked immunosorbent assay (ELISA). Fibrinogen activity was measured on the STA-R analyzer according to methods of Clauss. Von Willebrand factor (VWF) antigen was measured with the immunoturbidimetric method, using the STA Liatest kit (rabbit anti-hum VWF antibodies), following the instructions of the manufacturer. Measurement of antithrombin and protein C levels was performed with a chromogenic assay on the STA-R analyser. Free protein S was measured by an immune-turbidimetric method (Diagnostica Stago) accordingly to the manufacturer instructions. TFPI activity in plasma was measured by a chromogenic assay using the ACTICHROME TFPI activity assay (Sekisui Diagnostics, Stamford, Connecticut, USA) following the instructions of the manufacturer. TFPI activity was measured by inhibition of cleavage of a chromogenic substrate (spectrozyme Xa, Sekisui diagnostics) by factor Xa, after initiation of coagulation with an excess of factor X and Tissue Factor-Factor VIIa complex. D-dimer was assayed using the D-dimer HemosIL assay (Instrumentation Laboratory). The HemosIL D-Dimer HS is an automated latex enhanced immunoassay performed on the ACL TOP 700CTS (Instrumentation Laboratory, Warrington, UK). APC resistance was determined in samples from the MEGA study. APC resistance was measured with Cephotest (Nycomed Pharma, Oslo, Norway). The normalized APC sensitivity ratio (nAPCsr) was defined as the activated partial thromboplastin time (APTT) in the presence of APC divided by the APTT in the absence of APC in participants divided by the same ratio determined in normal pool samples, i.e. (APTT + APCparticipants / APTT - APCparticipants) / (APTT + APCnormalpool / APTT – APCnormalpool). White blood cell count, monocyte percentage, hemoglobin level, red cell distribution width, were measured using the Beckman coulter analyzer. hsCRP was measured in stored (at −80°C) and previously unthawed samples by automated particle-enhanced immunoturbidimetric assay (Tina-quant® CRP detection method; Roche Diagnostics, West Sussex, UK). Single nucleotide polymorphism (SNPs) were determined by PCR with the Taq-Man assay. The endogenous thrombin potential (ETP) was measured by calibrated automated thrombography (CAT). Thrombin generation was measured at 2 pM tissue factor. Coagulation was triggered by addition of 16 mM CaCl2, 30 µM phospholipids and measured by addition of 0.3 mM fluorogenic substrate, all final concentrations. ETP was calculated using the Thrombinoscope software (Thrombinoscope, Maastricht, The Netherlands).

APC resistance was determined in samples from the MEGA

study. APC resistance was measured with Cephotest (Nycomed

Pharma, Oslo, Norway). The normalized APC sensitivity ratio

(nAPCsr) was defined as the activated partial thromboplastin

time (APTT) in the presence of APC divided by the APTT in

the absence of APC in participants divided by the same

ratio determined in normal pool samples, i.e. (APTT +

APC

participants

/APTT ) APC

participants

) / (APTT + APC

nor-

malpool/A P T T ) APCnormal pool

).
