# Supplementary material for: Prediction of recurrent venous thrombosis in all patients with a first venous thrombotic event: The Leiden Thrombosis Recurrence Risk Prediction model (L-TRRiP)
Source: PLoS Med. 2019 Oct 11;16(10):e1002883. doi: 10.1371/journal.pmed.1002883 (PMC6788686; doi:10.1371/journal.pmed.1002883)
Supplement: S1 Protocol — MEGA, Multiple Environment and Genetic Assessment of Risk Factors for Venous Thrombosis. (PDF) [file pmed.1002883.s004.pdf]

Protocol
MEGA STUDIE

Multiple Environmental and Genetic Assessment of risk factors for venous thrombosis

NHS project: Veneuze trombose: gecombineerd effect van veel voorkomende erfelijke- en omgevingsfactoren

Prof. Dr F.R. Rosendaal
Afd. Klinische Epidemiologie
Afd. Trombose en Hemostase
Gebouw 1, CO-P
Leids Universitair Medisch Centrum
071 - 5264037

22 december 1998

Achtergrond

Veneuze trombose: gecombineerd effect van veel voorkomende erfelijke- en omgevingsfactoren

Veneuze trombose is een ziekte met een hoge incidentie. Een trombosebeen of longembolie komt jaarlijks voor bij 1 op de 1000 mensen. De ziekte brengt een enorme ziektelast met zich mee, vanwege de kans op blijvende klachten van het been (bij een trombosebeen) of van de luchtwegen (bij een longembolie). Eén tot twee procent van de tromboses verloopt zelfs dodelijk. Er zijn inmiddels verschillende risicofactoren bekend, zowel erfelijke- als omgevingsfactoren. De 'Leiden Thrombophilia Study' (LETS), een patiënt-controle onderzoek onder 948 personen heeft hieraan een grote bijdrage geleverd, door onder meer naar erfelijke afwijkingen van de bloedstolling te kijken. Factor V Leiden, een mutatie in het factor V gen, blijkt nu een van de meest voorkomende erfelijke risicofactoren te zijn voor het krijgen van een veneuze trombose. Een mutatie in het prothrombine gen is tevens een belangrijke risicofactor gebleken. De reeds langer bekende risicofactoren met betrekking tot de bloedstolling, antithrombine en proteïne C deficiëntie, bleken ook in LETS een verhoogd risico te geven. Naast erfelijke risicofactoren zijn er ook omgevings risicofactoren bekend. Te denken valt aan operaties, bedlegerigheid, zwangerschap en kraambed, pilgebruik en kwaadaardige ziekten.
Het is de laatste jaren duidelijk geworden dat een combinatie van zowel erfelijke- als omgevings risicofactoren tegelijkertijd een hoog risico kunnen geven op veneuze trombose. Dit blijkt voornamelijk uit familieonderzoeken, waarin vaak meerdere erfelijke afwijkingen in een persoon tegelijkertijd worden gevonden. Ook de aanwezige combinatie van een erfelijke- en omgevingsfactor geven een verhoogd risico: factor V Leiden in gelijktijdige aanwezigheid van het gebruik van de pil verhoogt de kans op trombose sterk. Van risicofactoren die veel voorkomen in de algemene bevolking kunnen wij verwachten dat zij gecombineerd in één persoon vaak voorkomen. Zij geven waarschijnlijk een belangrijke bijdrage aan het totale risico op een trombose. Op die combinatie van risicofactoren richt ons onderzoek zich.
Wij willen een patiënt-controle onderzoek gaan uitvoeren onder 2500 personen met een eerste trombose of longembolie en 2500 controlepersonen. Bij beide groepen willen wij de aan- of afwezigheid van een aantal frequent voorkomende erfelijke- en omgevings risicofactoren onderzoeken, zoals factor V Leiden, de protrombine mutatie, hyperhomocysteinemie, hoog factor VIII, operaties, immobilisaties, zwangerschap, kraambed, kwaadaardige ziekten en pilgebruik. Vervolgens willen wij het risico nagaan van personen die combinaties van deze risicofactoren hebben, en dit vergelijken met het risico van personen waarbij geen of slechts één risicofactor voorkomt. Wanneer voor verschillende combinaties van risicofactoren het risico goed bekend is, en wanneer dit voor specifieke combinaties hoog blijkt, zijn daarmee individuen gekarakteriseerd met een grote kans op ziekte. Dit zou vervolgens kunnen leiden tot preventie, met name gericht op omgevingsfactoren bij hen die al één of meerdere erfelijke risicofactoren hebben.
Het doel van deze MEGA studie is de effecten van gecombineerde risicofactoren en de kans op het krijgen van een trombosebeen, tromboseearm of longembolie na te gaan en dit kan alleen worden onderzocht in een groot aantal onderzoekspersonen. Dit patiënt-controle onderzoek zal dan ook het grootste onderzoek naar oorzaken van veneuze trombose zijn dat ooit is uitgevoerd.

Opzet

Deze MEGA studie is opgezet als een patiënt-controle onderzoek. Hierin zullen 2500 patiënten die een eerste trombosebeen, trombosearm of een longembolie hebben gehad, worden opgenomen. Tevens zullen 2500 controlepersonen worden geïncludeerd. Door de verdeling van gecombineerde risicofactoren te vergelijken tussen deze twee groepen, kan bepaald worden wat het risico op veneuze trombose is van degene met gecombineerde risicofactoren ten opzichte van degene zonder, of slechts met één risicofactor.
Dergelijke patiënt-controle onderzoeken hebben voorheen ook vanuit de afdeling Klinische Epidemiologie plaatsgevonden. Het reeds vermelde LETS onderzoek is uitgevoerd in samenwerking met de sectie Hemostase en Tromboseonderzoek van het LUMC en verschillende trombosediensten uit de randstad (goedkeuring medisch ethische commissie ref P149/89-9/DPE/cvdm). Tevens heeft een patiënt-controle onderzoek naar genetische variaties in stollingsparameters en het risico op een myocardinfarct plaatsgevonden, in samenwerking met de sectie Trombose en Hemostase en de afdeling cardiologie van het LUMC en de afdeling cardiologie van het Diaconessenhuis (goedkeuring medisch ethische commissie ref P83/93-5/DPE/EB). Beide zijn succesvol verlopen en de voorliggende studie is qua opzet en uitvoering gemodelleerd naar die eerdere projecten.

Selectie onderzoekspersonen

Patiënten worden gerecruteerd vanuit verschillende trombosediensten. Alle patiënten die vanaf de startdatum aangemeld worden met een eerste episode van een trombosebeen, trombosearm of longembolie, komen in principe voor dit onderzoek in aanmerking. Tevens worden de partners uitgenodigd als controlepersonen aan dit onderzoek deel te nemen.

Inclusiecriteria patiënten
- eerste objectief gediagnostiseerde diep veneuze trombose (CBO consensus, december 1998 Utrecht) na startdatum óf
- eerste objectief gediagnostiseerde longembolie (CBO consensus, december 1998 Utrecht) na startdatum
- leeftijd ten tijde van event < 80 jaar
- ‘antistollingsbehandeling in een trombosedienst (Leiden, Den Haag, Rotterdam, Utrecht, Amsterdam)

Exclusiecriteria patiënten
- veneuze trombose vóór startdatum van het onderzoek

Inclusiecriteria controles
- leeftijd < 80 jaar

Exclusiecriteria controles
- veneuze trombose vóór startdatum van het onderzoek

Van degenen die overleden zijn wordt voor een aantal variabelen via de huisarts gegevens verzameld (onder andere met betrekking tot maligniteiten).

Praktische uitvoering onderzoek

Identificatie patiënten
Patiënten zullen geïdentificeerd worden via de trombosediensten Leiden, Den Haag, Rotterdam, Utrecht en Amsterdam en eventueel meer participerende diensten, waar zij antistollingsbehandeling krijgen. De studie zal beperkt worden tot mannen als vrouwen onder de 80 jaar. In de trombosediensten wordt eens per twee weken / maandelijks een lijst gegenereerd met patiënten die mogelijk voor het onderzoek in aanmerking komen. De patiënt wordt in eerste instantie aangeschreven vanuit de trombosedienst. Vanuit het project wordt hierbij administratieve ondersteuning geleverd.

Uitnodiging deelname onderzoek
In de eerste brief wordt de patiënt schriftelijk geïnformeerd over het doel en de uitvoering van het onderzoek. Tevens wordt gevraagd of hij/zij, zijn/haar eventuele partner als controlepersoon zou willen uitnodigen voor dit onderzoek (bijlage 1). Deze brief wordt verstuurd ca. 4 weken na de trombose. Enkele weken later worden zij telefonisch benaderd, waarin nadere informatie over de MEGA studie, indien nodig, gegeven wordt. Indien patiënt en partner mee willen werken aan het onderzoek, krijgen zij elk een vragenlijst thuis gestuurd. Tevens wordt aan de patiënt gevraagd een toestemmingsformulier te ondertekenen. Hierin geeft hij aan ermee accoord te gaan dat medische gegevens met betrekking tot trombosebeen, trombosearm of longembolie worden opgevraagd bij de afdeling in het ziekenhuis waar hij vanwege de trombose opgenomen was. Indien geen ziekenhuisopname heeft plaatsgevonden, worden medische gegevens met betrekking tot de trombose of gerelateerde medische zaken (bv maligniteiten) bij de huisarts opgevraagd (bijlage 2). Het opvragen van deze gegevens is nodig om de diagnose, eerste trombosebeen, trombosearm of longembolie, te kunnen verifiëren. Vragenlijst(en) en toestemmingsformulier worden in een retourenvelop teruggestuurd naar Leiden. Zowel patiënt als partner wordt, ieder afzonderlijk, nadrukkelijk de mogelijkheid geboden van medewerking af te zien.

Interview
Vijf maanden na de start van antistolling (of 8 maanden indien een longembolie plaatsgevonden heeft), wordt in de trombosedienst nagegaan of en zoja, wanneer, de patiënt met de antistolling gestopt is. Een eventueel overlijden wordt geregistreerd, om te voorkomen dat deze persoon persoonlijk benaderd wordt voor het verdere onderzoek.
Drie maanden na het stoppen van de antistollingsbehandeling worden zowel patiënt als controlepersoon telefonisch uitgenodigd voor een interview. De afspraak wordt schriftelijk bevestigd. Tijdens het interview, dat plaats vindt in de trombosedienst, worden lengte en gewicht gemeten. Op dit moment wordt de deelnemers gevraagd een informed consent te tekenen waarin zij aangeven op de hoogte te zijn van het doel van het onderzoek en toestemming te geven voor het gebruiken van de gegevens verkregen uit vragenlijst, interview en venapunctie voor de MEGA studie (bijlage 3).

Gegevens uit interview en vragenlijst
De te verkrijgen gegevens uit interview en vragenlijst hebben onder meer betrekking op de medische voorgeschiedenis, voor zover deze van belang is. Zo zal er bijvoorbeeld gevraagd worden naar de aan- of afwezigheid van kwaadaardige ziekten en eventuele operaties en immobilisaties. Aan de patiënt zullen ook vragen gesteld worden over het trombosebeen, trombosearm of longembolie. Naast medische gegevens zal ook naar de aan- of afwezigheid

van mogelijke omgevings risicofactoren geinformeerd worden. Dit betreft onder andere rookgewoonten en alcoholgebruik. Een aantal vragen hebben alleen betrekking op vrouwen: zwangerschap, kraambed, pilgebruik en hormoon gerelateerde therapie.
Aan het einde van het interview zal een venapunctie plaats vinden. Naar verwachting zal het interview en de venapunctie circa 30 minuten per patiënt en controlepersoon duren.

Laboratoriumanalyses
Het bloed wordt door de interviewer getransporteerd naar het laboratorium in Leiden, alwaar het door een laborant verder verwerkt wordt. Het citraatbloed wordt afgedraaid en het verkregen plasma uitverdeeld over een aantal buisjes. Zo wordt ook serum en de inhoud van een Stabilytebuis over twee buisjes verdeeld. Het erfelijke materiaal wordt uit de witte bloedcellen geisoleerd. In eerste instantie worden deze buisjes opgeslagen in de vriezer. De laboratoriumanalyses vinden vervolgens batchgewijs, maanden tot mogelijk zelfs een jaar na de venapunctie plaats, om zo efficiënt mogelijk te werken. In plasma worden homocysteine waarden gemeten met behulp van de HPLC en factor VIII levels door middel van een ELISA. In het erfelijke materiaal worden de veelvoorkomende afwijkingen factor V Leiden en de protrombine mutatie (FII20210A) bepaald met behulp van PCR methoden. Als in de komende jaren efficiëntere methoden voor de bepalingen ter beschikking komen (bv multiplex PCR), willen we daar graag gebruik van maken. Indien de komende jaren een veelvoorkomende afwijking bekend wordt, ligt het in onze bedoeling het risico van trombose in aanwezigheid van deze afwijking (in combinatie met andere risicofactoren) na te gaan.

Statistische analyses
Karakteristieken van patiënten en controles, zoals leeftijd en rookgewoonten, zullen vergeleken worden, evenals de waarden van plasmabepalingen en de frequentie van afwijkingen tusen de twee groepen. Eventuele verschillen zullen getoetst worden met behulp van statistische analyses als de t-toets, analyses of variances, chi-kwadraat toetsen en dergelijke. Het risico op veneuze trombose in aanwezigheid van een risicofactor ten opzichte van de afwezigheid ervan zal worden berekend met behulp van een odds ratio. De bijbehorende 95% betrouwbaarheids-intervallen worden berekend volgens de methode van Woolf. Correcties voor eventuele confounders vindt plaats met behulp van logistische regressie. Om na te gaan of er interactie tussen twee risicofactoren is, vindt stratificatie naar de aan- en afwezigheid van beide risicofactoren plaats. Vervolgens worden odds ratios en betrouwbaarheidsintervallen berekend ten opzichte van de groep personen die geen van beide risicofactoren heeft. Interactie wordt gedefinieerd op grond van supra-additieve effecten. Er zal geen formele toetsing op interactie plaatsvinden. Bovenstaande analyses zullen herhaald worden voor specifieke subgroepen.

Codering van gegevens

Elke deelnemer ontvangt een codenummer. Deze codenummers zullen in de vragenlijsten worden gebruikt. Eén bepaalde bladzijde van de vragenlijst laat tevens het codenummer met naam, adres en geboortedatum van de persoon zien, om verwisselingen tijdens het invullen van de vragenlijst te voorkomen. Deze bladzijde wordt verwijderd zodra de vragenlijst bij de onderzoekers binnenkomt en vernietigd zodra de gegevens in een apart naam en adressenbestand gecontroleerd zijn. Dit bestand wordt beheerd door datamanagement en verder niet voor het onderzoek gebruikt, dan alleen voor het terugrapporteren naar de

deelnemers indien zij dit zelf wensen. De vragenlijst- en interviewgegevens worden gecodeerd verwerkt.
Tijdens de venapunctie krijgt elke deelnemer tevens een laboratoriumnummer, dat puur voor laboratoriumdoeleinden wordt gebruikt. Op de bloedmonsters komen dus geen namen of geboortedata van personen te staan. Uit de labnummers is niet af te leiden of het bloedmonsters van een patiënt of controlepersoon betreft. Op een apart formulier wordt de naam van de persoon en het bijbehorende labnummer vermeld. Dit formulier wordt doorgegeven aan datamanagement die dit nummer zal koppelen aan het adressenbestand om eventueel terug te kunnen rapporteren naar de deelnemers. Ook wordt het laboratoriumnummer gekoppeld aan de codenummers uit de vragenlijst- en interviewgegevens.

Rapportage einduitslagen

De deelnemende personen worden, als zij dit willen, op de hoogte gesteld van hun eigen resultaten. Het betreft uitslagen van die bloedbepalingen die vermeld staan onder 'laboratoriumanalyses'. Deze resultaten kunnen soms pas een jaar na bloedafname worden verstuurd. De reden hiervoor is dat de bloedbepalingen omwille van efficiëntie in grote aantallen tegelijkertijd worden uitgevoerd. Indien deelnemers aangeven graag zo spoedig mogelijk op de hoogte te worden gesteld van de aan- of afwezigheid van een afwijking, zoals bijvoorbeeld factor V Leiden, worden zij doorverwezen naar de eigen behandeld arts of huisarts. Aan het eind van het onderzoek, dus meer dan 4 jaar later, sturen wij een samenvatting van de wetenschappelijke resultaten uit de MEGA studie naar alle deelnemers. Deelnemers kunnen in de vragenlijst aangeven géén op prijs te stellen op terugrapportage. Uitslagen van bloedbepalingen, waarvan wij nu (nog) niet weten of we ze gaan doen (bijvoorbeeld omdat ze nog niet bekend zijn), worden niet naar deelnemers verstuurd. Dit betekent dus tevens dat na het versturen van de samenvatting na ruim 4 jaar, zij niet meer op de hoogte worden gebracht van eventueel verder onderzoek aan het bloedplasma of het erfelijke materiaal. Dit zal ook nadrukkelijk aan de deelnemers worden medegedeeld.

Projectgroep en samenwerking

De MEGA studie wordt uitgevoerd door de afdeling Klinische Epidemiologie van het Leids Universitair Medisch Centrum in samenwerking met onder meer de sectie Hemostase en Tromboseonderzoek (vakgroep Hematologie), waar het merendeel van de bepalingen zal worden verricht. Het Leyenburgziekenhuis uit Den Haag en het Radboud Ziekenhuis Nijmegen zullen de homocysteinebepalingen gaan verrichten. De trombosediensten uit Leiden, Den Haag, Rotterdam, Utrecht en Amsterdam verlenen hun medewerking aan dit onderzoek. De MEGA studie wordt gesubsidieerd door de Nederlandse Hartstichting. Recent hebben wij subsidie toegekend gekregen van het Koningin Wilhelmina Fonds. Dit project 'Kanker en het risico op veneuze trombose' geheten zal ten dele binnen deze MEGA studie worden uitgevoerd. Een protocol ter beoordeling hopen wij binnen enkele maanden aan de Medisch Ethische Commissie voor te leggen.

bijlage 1: uitnodigingsbrief (briefpapier trombosedienst)

MEGA studie
Klinische Epidemiologie
Gebouw 1, CO-P
Leids Universitair Medisch Centrum
Postbus 9600, 2300 RC Leiden
Tel: 071 – 526xxxx Fax: 071 – 5248122

. Naam
. Adres
. Postcode Woonplaats

________, datum
Betreft de MEGA studie

Geachte heer ________ / mevrouw _______ _________,

De trombosedienst ________ doet, in samenwerking met andere trombosediensten, een onderzoek naar combinaties van factoren die mogelijk het optreden van een eerste trombose of longembolie kunnen verklaren. Wij willen u vragen mee te werken aan dit onderzoek. De resultaten van dit onderzoek zijn vooral van belang om in de toekomst te kunnen voorkomen dat bepaalde mensen een trombose krijgen.

Bijgaand vindt u een uitgebreid informatiefolder over deze 'MEGA studie'. Indien u aan het onderzoek mee wilt doen, sturen wij u een vragenlijst. De vragenlijst kunt u thuis invullen en terugsturen. Drie maanden nadat u met antistolling gestopt bent willen wij u vragen naar de trombosedienst te komen. De rest van het onderzoek vindt daar plaats. Eén van de onderzoeksmedewerkers neemt dan de ingevulde vragenlijst met u door. Uw lengte en gewicht worden gemeten. Ook wordt een geringe hoeveelheid bloed afgenomen, waarvoor u nuchter moet komen. Het onderzoek vindt plaats in de ochtend, is éénmalig en duurt ongeveer 30 minuten. Ook voor een vergelijkingsgroep zoeken wij mensen. Indien u een partner heeft, zou deze daar aan kunnen meewerken.

Uw besluit om wel of niet mee te doen aan dit onderzoek heeft vanzelfsprekend geen enkel gevolg voor uw behandeling. De behandeling door uw huisarts of specialist gaat gewoon door. Alle gegevens vallen onder het medisch beroepsgeheim en worden strikt vertrouwelijk behandeld.

De afdeling Klinische Epidemiologie van het Leids Universitair Medisch Centrum te Leiden coördineert het onderzoek. Eén van onze onderzoeksmedewerkers neemt tijdens kantooruren binnenkort telefonisch contact met u op. Mocht u nog vragen over het onderzoek hebben dan kunt u die aan de medewerker stellen. Indien u tijdens kantooruren

thuis niet telefonisch bereikbaar bent, kunt u zelf bellen naar _____ op ___ tussen ___ en __ op telefoonnummer 071___. Bij voorbaat hartelijk dank.

Met vriendelijke groeten, namens de onderzoeksgroep,

Dr ________
Medisch Directeur
Trombosedienst _____

Drs C.J.M. Doggen
Projectleidster MEGA studie
Afdeling Klinische Epidemiologie
Leids Universitair Medisch Centrum

Dit onderzoek wordt mede mogelijk gemaakt door de Nederlandse Hartstichting (logo.

Informatiefolder MEGA studie

Veneuze trombose: gecombineerd effect van veel voorkomende erfelijke- en omgevingsfactoren

Achtergrond

Een trombosebeen of trombosearm wordt veroorzaakt door een afsluiting van een bloedvat in het been of de arm door een bloedstolsel. Soms kan een bloedstolsel uit een been of arm losschieten en naar de longen gaan. Als een stolsel in de longen een vat afsluit kan een longembolie ontstaan.

Een bloedstolsel kan door verschillende aanleidingen ontstaan. Een operatie, bijvoorbeeld, geeft een licht verhoogde kans op trombose. Maar lang niet iedereen die een operatie ondergaat krijgt een trombose. Mogelijk zijn er meerdere aanleidingen tegelijkertijd nodig om een bloedstolsel te laten ontstaan. Dit zou kunnen verklaren waarom de ene persoon wel, en de andere geen trombose of longembolie krijgt. Hier zouden wij graag onderzoek naar doen in de MEGA studie.

In dit onderzoek willen wij niet alleen weten of iemand een operatie heeft gehad. Wij zijn ook geinteresseerd in langdurige bedrust, pilgebruik, zwangerschap en dergelijke. Tevens willen we nagaan of afwijkingen in het bloed een rol spelen in het ontstaan van een trombose of longembolie. Wij kijken hierbij onder andere naar het erfelijk materiaal van stoffen die het bloed helpen stollen. Hiervoor willen wij een kleine hoeveelheid bloed af nemen. De resultaten van dit onderzoek zijn vooral van belang om in de toekomst te kunnen voorkomen dat bepaalde mensen een trombose krijgen.

Wie worden voor de MEGA studie uitgenodigd?

Voor dit onderzoek nodigen wij mensen uit die een trombosebeen, trombosearm of longembolie hebben gehad. Daarnaast willen wij deze groep mensen vergelijken met een zogenaamde vergelijkingsgroep. Dit is een willekeurige steekproef uit de bevolking. In deze vergelijkingsgroep willen wij hetzelfde onderzoeken als bij de mensen die een trombose of longembolie hebben gehad. Indien u een partner heeft willen wij deze daar voor uitnodigen.

Hoeveel mensen worden uitgenodigd en hoe lang loopt het onderzoek?

Dit wordt het grootste onderzoek naar trombose dat ooit gedaan is. Voor het slagen van dit onderzoek is het belangrijk dat 5000 mensen mee gaan werken. De ene helft bestaat uit mensen die een trombosebeen, trombosearm of longembolie hebben doorgemaakt. De andere helft bestaat uit de vergelijkingsgroep. In de loop van 4 jaar zullen mensen voor deelname aan de MEGA studie worden uitgenodigd.

Krijgen deelnemers resultaten van de MEGA studie?

Mensen die de uitslag van hun eigen bloedbepalingen willen krijgen dit in de loop van het onderzoek toegestuurd. Dit kan enige tijd duren. Soms kunnen pas een jaar later persoonlijke resultaten toegestuurd worden. De reden hiervoor is dat de bloedbepalingen in grote aantallen tegelijkertijd zullen worden gedaan. Aan het eind van het onderzoek,

dus meer dan 4 jaar later, sturen wij een samenvatting van de resultaten uit de MEGA studie naar alle deelnemers.

Heeft deelname aan de MEGA studie gevolgen voor mijn behandeling? Worden mijn gegevens anoniem verwerkt?

Wel of geen deelname aan dit onderzoek heeft geen enkel gevolg voor uw eventuele behandeling. Wij veranderen ook niets aan uw behandeling. Als u vragen heeft omtrent uw eigen trombose of longembolie kunt u zoals altijd bij uw behandelend specialist of huisarts terecht. Alle gegevens die uit dit onderzoek verkregen worden vallen onder het medisch beroepsgeheim. Deze gegevens worden strikt vertrouwelijk behandeld. Uw naam en adres komen niet op de vragenlijst of bloedmonsters voor. Hier staan wel nummers op die alleen door de onderzoek medewerkers in Leiden aan uw naam en adres gekoppeld kunnen worden.

VITRO onderzoek

Misschien hoort u tot de groep mensen die door de onderzoekers van het VITRO onderzoek benaderd zijn. Dit onderzoek staat los van deze MEGA studie en onderzoekt een ander aspect van trombose. De onderzoekers van beide studies werken nauw samen. U kunt zelf beslissen of u aan één of beide onderzoeken deelneemt. Beide onderzoeken kunnen tesamen een duidelijker beeld geven over trombose.

Als ik nog vragen heb over het onderzoek?

Wij hopen dat u mee wilt werken aan deze MEGA studie. Voor vragen omtrent het onderzoek kunt u tijdens kantooruren bellen naar de medewerkers ________ of ______ van de afdeling Klinische Epidemiologie in Leiden op telefoonnummer: 071 – 526 ______.

Dit onderzoek wordt mede mogelijk gemaakt door Nederlandse Hartstichting (logo).
